# Opportunities for biodiversity gains under the world's largest reforestation programme

**DOI:** 10.1038/ncomms12717

**Published:** 2016-09-06

**Authors:** Fangyuan Hua, Xiaoyang Wang, Xinlei Zheng, Brendan Fisher, Lin Wang, Jianguo Zhu, Ya Tang, Douglas W. Yu, David S. Wilcove

**Affiliations:** 1 Program in Science, Technology, and Environmental Policy, Woodrow Wilson School of Public and International Affairs, Princeton University, Princeton, NJ 08544, USA; 2 State Key Laboratory of Genetic Resources and Evolution, Kunming Institute of Zoology, Chinese Academy of Sciences, Kunming, Yunnan 650223, China; 3 Kunming College of Life Sciences, University of Chinese Academy of Sciences, Kunming, Yunnan 650223, China; 4 College of Architecture and Environment, Sichuan University, Chengdu, Sichuan 610000, China; 5 Gund Institute for Ecological Economics, Rubenstein School of Environment and Natural Resources, University of Vermont, Burlington, VT 05405, USA; 6 School of Biological Sciences, University of East Anglia, Norwich Research Park, Norwich, Norfolk NR47TJ, UK; 7 Department of Ecology and Evolutionary Biology, Princeton University, Princeton, NJ 08544, USA

## Abstract

Reforestation is a critical means of addressing the environmental and social problems of deforestation. China's Grain-for-Green Program (GFGP) is the world's largest reforestation scheme. Here we provide the first nationwide assessment of the tree composition of GFGP forests and the first combined ecological and economic study aimed at understanding GFGP's biodiversity implications. Across China, GFGP forests are overwhelmingly monocultures or compositionally simple mixed forests. Focusing on birds and bees in Sichuan Province, we find that GFGP reforestation results in modest gains (via mixed forest) and losses (via monocultures) of bird diversity, along with major losses of bee diversity. Moreover, all current modes of GFGP reforestation fall short of restoring biodiversity to levels approximating native forests. However, even within existing modes of reforestation, GFGP can achieve greater biodiversity gains by promoting mixed forests over monocultures; doing so is unlikely to entail major opportunity costs or pose unforeseen economic risks to households.

## Abstract (web summary)

China's Grain for Green Program is the world's largest reforestation program, encompassing tens of millions of hectares since 1999. Here, Hua *et al*. show that the majority of areas have been reforested with tree monocultures, but that planting mixed forests could increase animal biodiversity without imposing additional economic costs.

## Graphical abstract

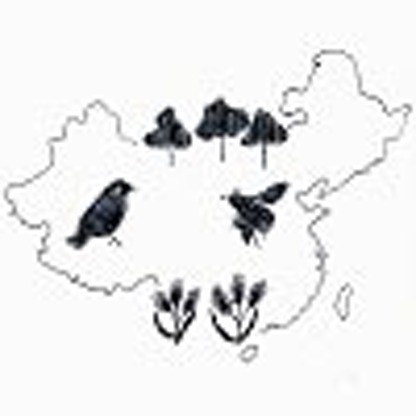

Reforestation programs are a widely used policy instrument for reversing the environmental and livelihood problems created by deforestation and climate change^1^,^2^,^3^,^4^. China's nationwide Grain-for-Green Program (GFGP) is the largest reforestation programme in the world^5^,^6^,^7^. Initiated in 1999 primarily to control soil erosion^8^, GFGP uses cash payments to incentivize rural households to reestablish forest, shrub and/or grassland on sloped cropland and scrubland^6^,^9^,^10^. Government statistics show that as of 2013, GFGP has reestablished 27.8 million hectares (ha) of forest in 26 of China's 31 mainland provinces, with government spending of US$ 46.91 billion (calculated at an exchange rate of 6.41 CN¥ US$^−1^ in November 2015)^11^. The Chinese government has committed to extending GFGP until at least 2020, with planned retirement of an additional 2.83 million ha of marginal cropland (towards reforestation as well as restoration of shrub and grassland)^12^. GFGP forests are expected to stay forested beyond the conclusion of the programme^12^.

The vast majority of GFGP forests are intended to be used for production of timber, tree fruits and other cash crops, with biodiversity restoration only a secondary consideration^8^,^12^. However, GFGP's enormous scale dictates that it should have profound effects on China's biodiversity^7^,^13^ and potentially offer substantial biodiversity co-benefits^14^, especially since most of the forests will be subject to periodic harvesting and re-planting, which opens up the possibility of changing forest cover across tens of millions of hectares. It is therefore important to understand if opportunities exist for improving biodiversity conservation under GFGP. This has remained a strikingly unexplored issue^6^,^9^,^15^ despite growing concerns regarding the nature of reforestation under GFGP and its biodiversity implications^5^,^16^.

Here we report a two-part study that explores the opportunities for biodiversity conservation under GFGP. First, we extensively examined the peer-reviewed literature to synthesize information on the tree composition of GFGP forests across China. Notwithstanding concerns over the nature of GFGP forests^6^,^16^,^17^,^18^, there has yet to be a nationwide synthesis of the modes of reforestation based on tree species composition; such a synthesis is essential to understanding GFGP's biodiversity implications. Second, we undertook a field study in south-central Sichuan Province to quantify GFGP's biodiversity impacts, the potential for biodiversity gains, and the opportunity costs of realizing such gains, using birds and bees (Hymenoptera, Anthophila) as representative taxa. Specifically, we focused on answering the three following questions: (1) how does the biodiversity of GFGP forests compare with the biodiversity of the croplands they are replacing? (2) How does the biodiversity of GFGP forests compare with the biodiversity of the native forests that characterized the land cover that preceded the croplands now being reforested? (3) What are the opportunity costs associated with planting different types of GFGP forest? This last question becomes important if some types of GFGP forest are deemed more desirable for biodiversity than other types. Answering these questions can provide an empirical assessment of the conservation opportunities under GFGP in this biodiverse region of China, and stands to inform similar assessments elsewhere in the country. Finally, we identified priorities for further scientific research and policy formulation related to GFGP and biodiversity.

We find that, across China, GFGP forests are overwhelmingly monocultures or compositionally simple mixed forests. In south-central Sichuan, GFGP reforestation using monocultures generally results in net losses of bird diversity while GFGP using mixed forest generally results in net gains; all current modes of GFGP reforestation result in overwhelming losses of bee diversity. Moreover, all current modes of GFGP reforestation fall well short of restoring biodiversity to levels approximating native forests that preceded the croplands now being reforested. There is thus considerable scope for biodiversity gains if GFGP were to incentivize the conservation and restoration of native forests over compositionally simple forests. Finally, even within existing modes of reforestation, GFGP can achieve biodiversity gains by promoting mixed forests over monocultures. Such a shift would benefit bird diversity and carries no penalty for bee diversity; in terms of forest production, it also is unlikely to carry opportunity costs or pose unforeseen economic risks to households.

## Results

### Composition of GFGP forests across China

**Figure 1:**
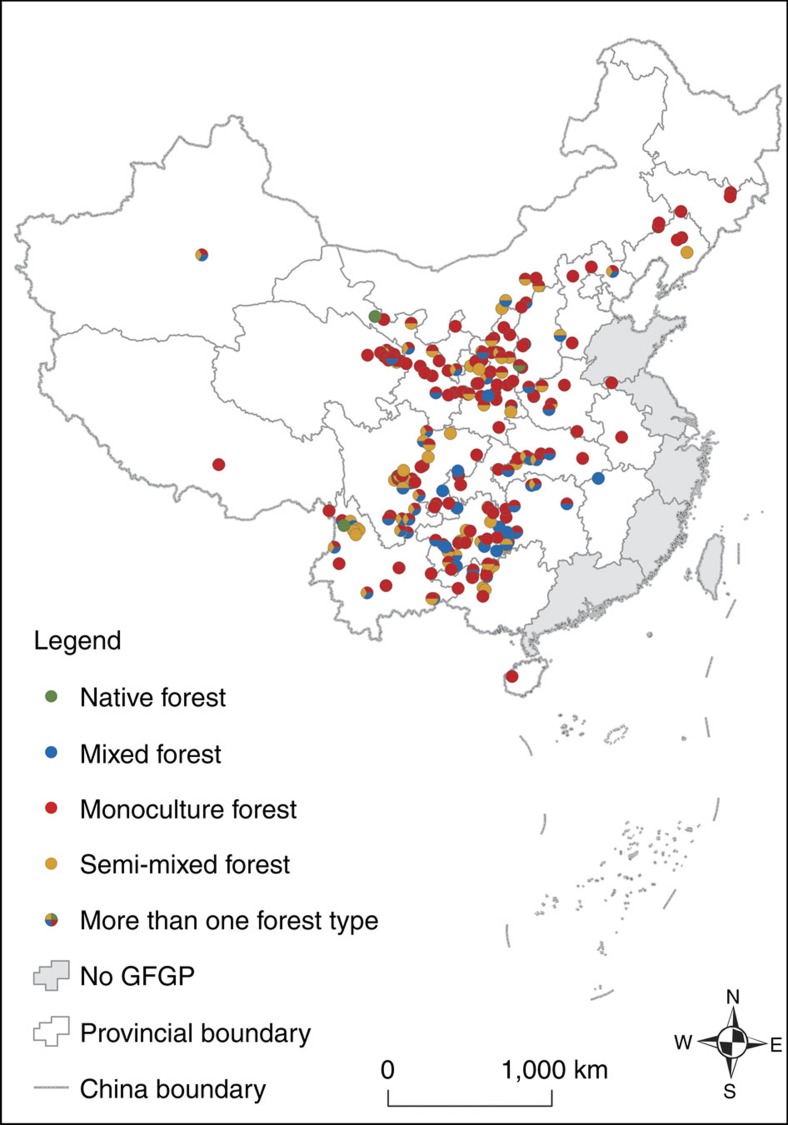
Distribution of different types of GFGP forests across China. Dots with different colours represent different types of GFGP forest: green, native forest; blue, mixed forest; red, monoculture forest and yellow, semi-mixed forest (that is, monoculture forest mixed with compositionally simple shrub or ground cover; not present in our study region in Sichuan); dots with more than one colour represent sites where more than one type of GFGP forest has been established. Provinces in grey are those where GFGP has not been implemented. See Supplementary Data 1 for detailed list of compiled literature. Administrative borders for China and individual provinces are courtesy of the National Geomatics Center of China^73^. Nature publications remain neutral with regard to contested jurisdictional claims in published maps.

Synthesis of 258 peer-reviewed English- and Chinese-language publications revealed that, while GFGP forests across China use a wide range of tree species (Supplementary Data 1), forest stands at individual locations are overwhelmingly monocultures and, to a lesser extent, compositionally simple mixed forests (that is, forests consisting of two to five tree species; Fig. 1). Out of 202 reported locations (all reported locations are counties except for a few cases where estimates were available only for municipalities, the next administrative unit above counties) from 23 provinces, monocultures were planted at 166 (82.2%) locations, monocultures with one or two shrub or grass species were planted at 72 (35.6%) locations, and mixed forests were planted at 78 (38.6%) locations. Only three locations reported planting native forest (Fig. 1; Supplementary Data 1; percentages do not sum up to 100% because more than one forest type can be planted in a single location).

### Overview of field study in south-central Sichuan

**Figure 2:**
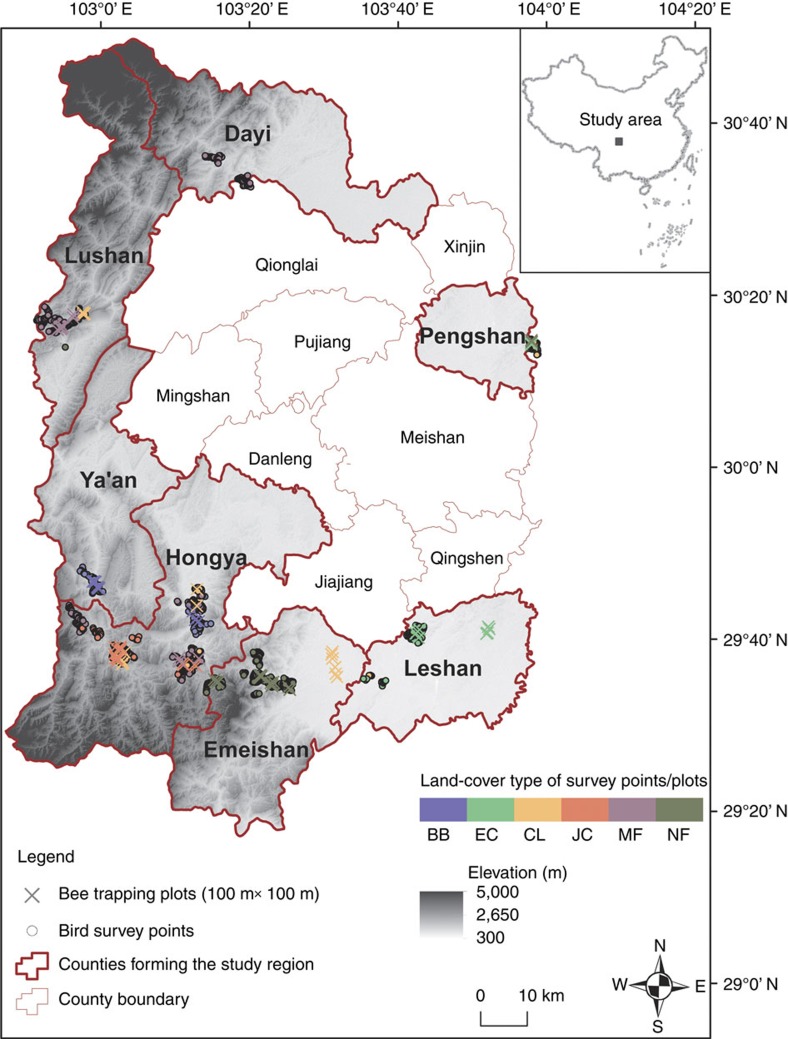
Locations of biodiversity sampling sites for different land-cover types in south-central Sichuan. Each polygon is a county, with the text inside indicating its name. Counties in bold and colour are where field surveys were conducted, with elevation displayed in a gradient of grey shades using the Digital Elevation Model (DEM) data courtesy of the Land Processes Distributed Active Archive Center^74^. Dot and cross signs represent sampling sites for birds (point count stations) and bees (trapping plots), respectively, with different colours representing different land-cover types: CL (yellow), cropland; NF (dark green), native forest; EC (light green), eucalyptus monoculture; BB (blue), bamboo monoculture; JC (red), Japanese cedar monoculture and MF (purple), mixed GFGP forest. Administrative borders for individual counties are courtesy of the National Geomatics Center of China^73^. Nature publications remain neutral with regard to contested jurisdictional claims in published maps.

To assess the biodiversity implications of GFGP, we focused on a region of 7,949 km^2^ in south-central Sichuan Province (Fig. 2). This region sits on the eastern edge of the south-central China biodiversity hotspot^19^ and spans an elevation range of 315–1,715 m. It was historically forested but suffered heavy deforestation starting in the 1950s (ref. 20). GFGP reestablished ∼54,800 ha of forest here from 1999 to 2014, mostly on sloped terrain and in contiguous expanses^11^; there has been no grassland restoration under GFGP in this region. Through pilot surveys, we identified four dominant types of forest reestablished under GFGP in this region: monocultures of (1) eucalyptus, (2) bamboo and (3) Japanese cedar, and compositionally simple (4) mixed forest consisting of two to five tree species (Supplementary Table 1). Mixed forests at different locations tended to vary in tree species composition but were similar in the small number of tree species they contained (see Supplementary Note 1 for species details for all types of GFGP forest). The generally small size of landholdings in this region (median=0.4 ha; see below and Supplementary Fig. 1) dictates that monocultures resulted predominantly from neighbouring households choosing to plant the same tree species, while mixed forests resulted mostly from neighbouring or individual households planting stands of different tree species (only about a quarter of mixed forest consisted of bona fide, individual-level mixtures of tree species; Supplementary Note 1). The vast majority of these GFGP forests are production forests, subject to repeated harvesting and re-planting; they thus constitute dynamic landscapes potentially open to new types of forest cover.

We evaluated GFGP's biodiversity impacts and potential biodiversity gains by comparing the biodiversity of different types of GFGP forest with that of two baseline land-cover types: cropland and native forest (Supplementary Table 1; Supplementary Note 1). We also evaluated the opportunity costs of realizing alternative biodiversity outcomes by analysing the production costs and profits of different types of GFGP forest. Sloped cropland, consisting of mixtures of rice, corn and vegetables and generally of low-agricultural intensity, is the land cover being reforested under GFGP in our study region. Native forest, specifically broadleaf, subtropical evergreen forest that has been subject to selective logging and other extractive uses for a long time, provides a benchmark of the extent to which GFGP restores biodiversity to presumably historical levels that preceded the croplands now being reforested (that is, before the Great Leap Forward Era in the late 1950s, which initiated large-scale forest conversion in China in the modern era^21^. It should be noted that this region has a human settlement history stretching back thousands of years^22^). We selected large expanses (≥60 ha) of the six target land-cover types (cropland, native forest, three GFGP monocultures and mixed GFGP forest) for biodiversity surveys. The general lack of cropland on sloped terrain (due largely to the success of GFGP) forced us to use croplands on flatter terrain. Compared with the sloped croplands that were reforested under GFGP, these flatter croplands are likely subject to more intensive farming as manifested by reduced amounts of hedgerows, shade trees and other non-farm vegetation^15^,^23^, and may therefore support less biodiversity^24^,^25^; alternatively, however, the flatter terrain could have more fertile soils, and thus support more biodiversity through higher ecosystem productivity^26^. The native forests we used were mostly concentrated around Emei Mountain and have been degraded by extensive timber and non-timber extraction. Because of the small size of individual landholdings in the region, our biodiversity surveys necessarily covered GFGP forest stands in different growth stages and with different management intensities, although we surveyed only forest stands with closed canopy. Because the rotation cycles for GFGP forests are generally short (typically six to seven years for eucalyptus and 18–20 years for Japanese cedar; Supplementary Note 1), the effects of differences in stand age on biodiversity patterns are likely to be weaker than would be the case in forest systems with long rotation cycles.

We focused on birds and bees as representative taxa. Birds are considered reasonably good indicators of the response of animal diversity to changes in physical vegetative structure^27^. Bees rely heavily on floral resources, particularly in the forest understory^28^, and therefore represent a complementary component of biodiversity that responds more strongly to the species composition of the forest understory; they are also important providers of pollination services. We conducted community surveys of birds and bees using point counts and plot-based pan trapping, respectively, in all six land-cover types, and used DNA barcoding to identify bee specimens to species level. Point-count data on birds during the non-breeding season were supplemented by data on mixed-species flocks because many bird species form mobile foraging flocks during the non-breeding season. To compare the biodiversity of GFGP forests with the biodiversity of cropland and native forest, we used both community-level metrics (species richness and community compositional turnover) and species-level metrics (species abundance and habitat specialization). We stratified the bird and bee surveys and analyses into elevation bands to minimize complications from the effects of elevation on biodiversity patterns. Based on their distribution patterns, surveys and analyses of eucalyptus, bamboo and Japanese cedar monocultures, cropland, and native forest were stratified into low, mid and high-elevation bands, while surveys and analyses of mixed forest were stratified into mid and high-elevation bands (Supplementary Table 2). We analysed the bird data using all three bands, but for the bee data, we pooled the mid and high elevation bands because of limited sample size. In all, for birds, we conducted 584 point counts over the breeding season (May–July) and 564 point counts over the non-breeding season (December) in 2014; for bees, we ran 74 trapping plots in May–July of 2014 (Fig. 2; Supplementary Table 3).

For economic analyses, we quantified the contribution of forest production to household income and compared forest production costs and profits among different types of GFGP forest, through semi-structured household interviews in villages around our biodiversity survey sites. We interviewed household heads for 30–40 min using a questionnaire that asked respondents to self-report the percentage of household income contributed by forest production, and that broke down and inquired about detailed aspects of the cost of and income streams from forest production (for example, seedling, fertilizer and/or labour cost during forest establishment, maintenance and selective/clear-cut harvest and so on). We calculated the average annual cost of and income from forest production per ha, factoring in forest harvest cycles and an annual discount rate of 5%. In all, we interviewed 166 households (≥35 households for each type of GFGP forest; Supplementary Table 4) in July of 2015.

### Biodiversity in GFGP forests versus cropland

**Figure 3:**
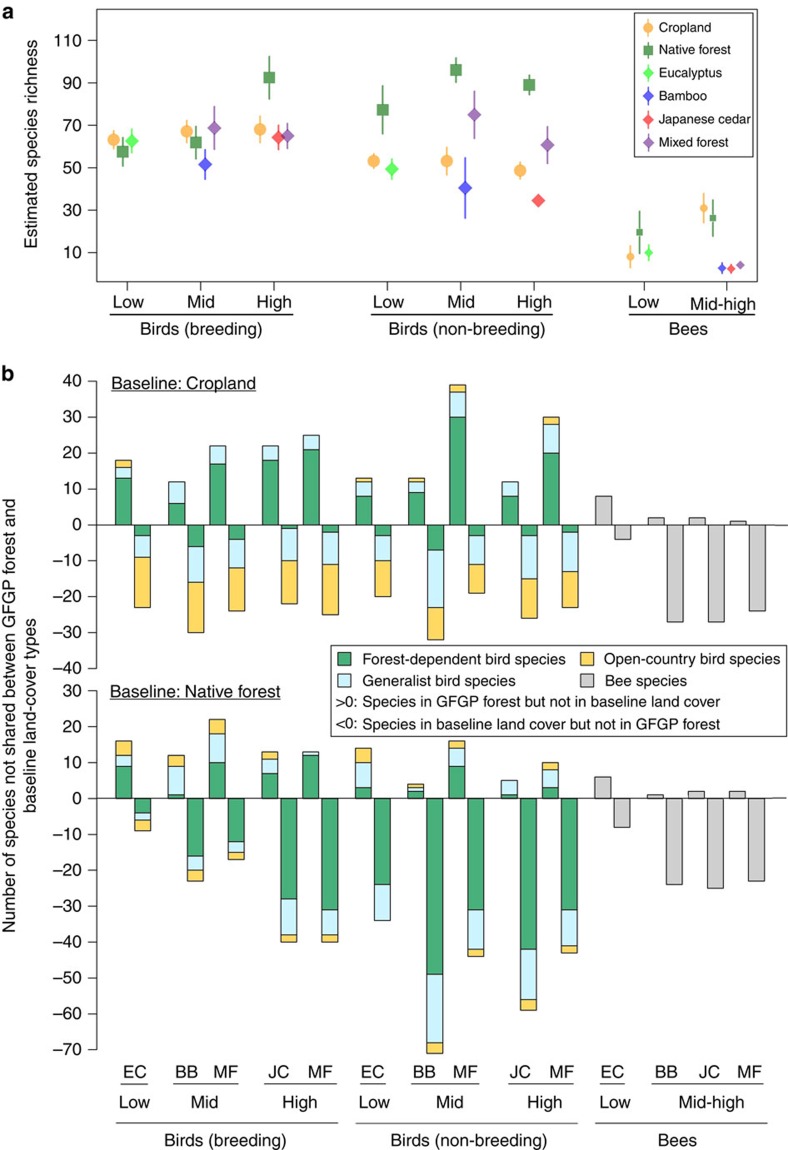
Biodiversity comparison between GFGP forests and baseline land-cover types. Comparisons are given in terms of community-level analyses, stratified into elevation bands (low, mid and high for birds; low and mid-high for bees). (**a**) Comparison of bird and bee species richness among GFGP forests, cropland and native forest, based on species accumulation curves. Symbols of different shapes and colours represent different land-cover types: yellow circle, cropland; dark green square, native forest; light green diamond, eucalyptus monoculture; blue diamond, bamboo monoculture; red diamond, Japanese cedar monoculture and purple diamond, mixed GFGP forest. Error bars represent 95% confidence intervals. Bee species richness is shown using smaller symbols than bird species richness in order to display the shorter error bars. (**b**) Community compositional turnover of GFGP forests relative to cropland (upper row) and native forest (lower row), visualized as the number of species not shared between each type of GFGP forest and the baseline land-cover types. Species above zero refer to those that were present in GFGP forests but not in the baseline land covers, and species below zero refer to those that were present in baseline land cover but not in GFGP forests. For birds, each species is placed within one of three habitat association guilds and colour coded accordingly: green, forest-dependent species; light blue, generalist species and yellow, open-country species (Supplementary Data 6). Bees are represented in grey. Abbreviations of GFGP forest type are as follows: BB, bamboo monoculture; EC, eucalyptus monoculture; JC, Japanese cedar monoculture and MF, mixed GFGP forest.

The bird species richness of mixed forest was 25–41% greater compared with cropland during the non-breeding season and similar during the breeding season (Fig. 3a). In contrast, the bird species richness of monocultures was generally similar to or lower than that of cropland in both the breeding and non-breeding seasons (Fig. 3a). Bee species richness was similar to cropland in eucalyptus forest but 87–92% lower in all other GFGP forests relative to cropland (Fig. 3a). For both bird and bee communities, reforestation resulted in considerable community compositional turnover (PERMANOVA *t*-test, *P*<0.05; Supplementary Fig. 2); this turnover was predominantly the result of forest-dependent species replacing open-country species in the case of birds, and of overwhelming species loss in the case of bees (Fig. 3b; Supplementary Data 2). We also compared the population abundances of shared species between each type of GFGP forest and cropland (generalized linear models within N-mixture models using a Poisson error distribution, *P*<0.05; *P* corrected using the Benjamini–Hochberg correction for multiple testing because multiple species were analysed^29^). Bird species shared between each type of GFGP forest and cropland generally had similar abundances in GFGP forests as in cropland; for bees, no shared species had higher abundances in GFGP forests than in cropland, while a number of species had lower abundances (Supplementary Table 5; Supplementary Data 3). In general, therefore, GFGP monocultures resulted in reduced diversity of both birds and bees compared with cropland; GFGP mixed forests harboured more bird species and similar numbers of birds compared with cropland, but were lacking in bees.

### Biodiversity in GFGP forests versus native forest

Compared with native forest, GFGP forests generally had 17–61% fewer bird species across both seasons and 49–91% fewer bee species; only occasionally did GFGP forests have comparable species richness to native forest (Fig. 3a). The lower bird species richness relative to native forests was especially pronounced in monocultures and during the non-breeding season (Fig. 3a). For both bird and bee communities, there was significant turnover in community composition between any type of GFGP forest and native forest (PERMANOVA *t*-test, *P*<0.05), with the notable exception of bird communities in mixed GFGP forest during the non-breeding season (Supplementary Fig. 3). Such turnover was predominantly the result of GFGP forests harbouring a subset of the species existing in native forest; notably, a number of forest-dependent bird species in native forest were absent from GFGP forests (Fig. 3b; Supplementary Data 2). As with cropland, we compared the population abundances of shared species between each type of GFGP forest and native forest (generalized linear models within N-mixture models using a Poisson error distribution, corrected *P*<0.05). Both bird and bee species shared between each type of GFGP forest and native forest generally had lower abundances in GFGP forests than in native forest (Supplementary Table 5; Supplementary Data 4). In summary, all types of GFGP forests harbour lower bird and bee diversity than do the degraded native forest that likely typified the pre-1950s land cover cleared to create the croplands that are now being reforested under GFGP.

### Biodiversity uniqueness of different land-cover types

**Figure 4:**
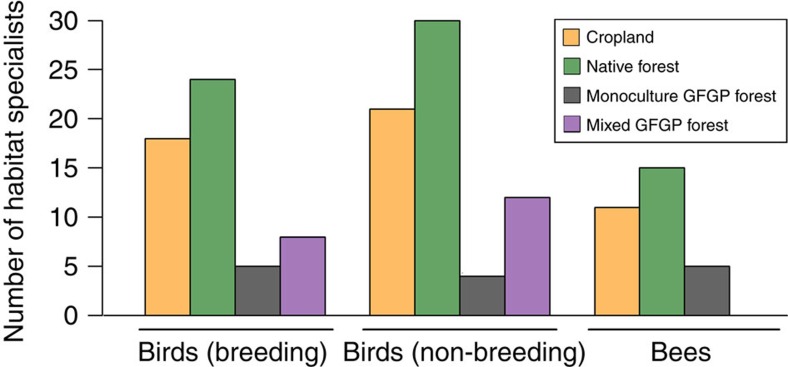
The number of habitat specialist species associated with each land-cover type. The three monoculture GFGP forests are represented as one ‘monoculture GFGP forest' category. Bars of different colours represent different land-cover types: yellow, cropland; dark green, native forest; black, monoculture and purple, mixed GFGP forest.

We tallied the number of habitat specialists, defined in our study as species found in only one land-cover type, to gauge the biodiversity uniqueness of each land-cover type (while acknowledging that some species may occur in habitats other than the ones we studied). Because each of the three types of GFGP monoculture forests covered only a small section of the whole elevation range we studied, which could limit the number of habitat specialists they contain, we combined them into one ‘monoculture' forest type to minimize this effect. For both birds and bees, native forest consistently had the most habitat specialists, followed by cropland (Fig. 4; Supplementary Data 5). GFGP forests, on the other hand, had comparatively few habitat specialists. Within GFGP forests, the number of habitat specialists was consistently higher in mixed forest than in monocultures for birds, but was low in all GFGP forests in the case of bees (Fig. 4; Supplementary Data 5).

### Biodiversity in mixed versus monoculture GFGP forests

In the case of birds, mixed forest was clearly more biodiverse than monocultures: it had higher species richness and more habitat specialists (Figs 3 and 4). Notably, while GFGP reforestation using monocultures generally resulted in net losses of bird diversity compared with cropland, GFGP reforestation using mixed forest generally resulted in net gains (Figs 3 and 4). However, for bees, mixed forest was similar to monocultures in terms of species richness and the number of habitat specialists (Figs 3 and 4). Mixed forest is thus more beneficial than monocultures for bird diversity and no worse than monocultures for bee diversity.

### Opportunity costs of more biodiverse mixed GFGP forests

**Figure 5:**
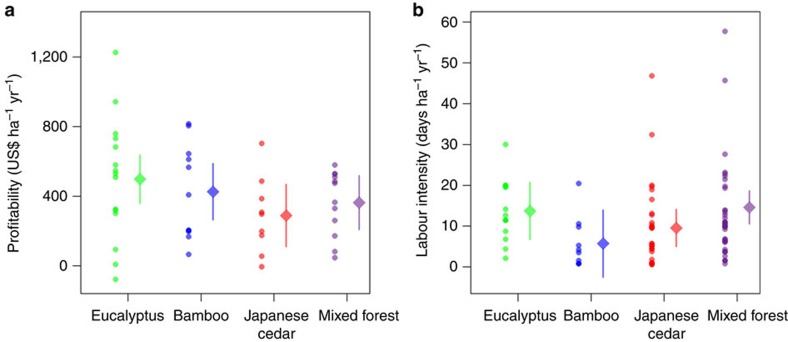
Economic comparison among different types of GFGP forests. Comparisons are given in terms of annual (**a**) profit and (**b**) labour intensity of forest production. Scattered dots and diamonds represent observed data and model estimates, respectively; error bars represent 95% confidence intervals.

We report economic analyses of net rent to forest production based on an in-depth survey of 166 households. Below we use a discount rate of 5% over the respective production cycles of different types of GFGP forests, but our results remain qualitatively unchanged under alternative discount rates (Supplementary Table 6). The regional median and mean percentages of annual household income contributed by forest production were 5 and 12.8%, respectively (Supplementary Fig. 1), corresponding to a profit of US$ 365.06 and 431.02 ha^−1^ per year, respectively. In terms of forest profit, labour intensity and the percentage of household income contributed by forest production (after accounting for the effect of forest area; Supplementary Fig. 1), mixed forest was similar to monocultures of eucalyptus, bamboo and Japanese cedar (multiple linear models, *P*>0.05; Fig. 5; Supplementary Table 7). Therefore, switching from monocultures to mixed forest is unlikely to carry opportunity costs, or pose unforeseen economic risks to households considering the generally low importance of forest production to household income.

## Discussion

Combining a literature review, biodiversity assessments of birds and bees, and economic analyses, we reach four conclusions regarding GFGP's biodiversity implications in south-central Sichuan Province. First, the forests planted through GFGP are overwhelmingly monocultures and compositionally simple mixed forests; almost none contain a diverse mixture of native trees. Second, GFGP reforestation using monocultures generally results in net losses of bird diversity while GFGP using mixed forest generally results in net gains; all current forms of GFGP reforestation result in overwhelming losses of bee diversity (Figs 3 and 4). Third, existing modes of GFGP reforestation fall well short of restoring biodiversity to levels approximating the native forests that preceded the croplands now being reforested (Figs 3 and 4). There is thus considerable scope for biodiversity gains if GFGP were to incentivize the conservation and restoration of native forests over structurally and compositionally simple forests. Even assuming no economic returns from native forest for the landholder (an extremely conservative assumption, given that native forest can generate a range of production and non-production incomes^1^,^30^), such a shift in reforestation goals would on average carry an opportunity cost of roughly 431 US$ ha^−1^ per year or 12.8% of household income. Finally, even within existing modes of reforestation, GFGP can achieve biodiversity gains by promoting mixed forests over monocultures (Figs 3, 4, 5). Such a shift would benefit bird diversity and carries no penalty with respect to bee diversity; in terms of forest production, it also is unlikely to carry opportunity costs or pose unforeseen economic risks to households.

These findings are consistent with current knowledge of forest biodiversity and the productivity of mixed versus monoculture forests, and with the socioeconomic context of south-central Sichuan. Our findings that mixed forests exceed monocultures in bird diversity and that native forest exceeds all GFGP forests in both bird and bee diversity echo the general rule that forests with higher compositional and structural diversity tend to harbour higher levels of biodiversity^31^,^32^. The very low bee diversity in all types of GFGP forests except eucalyptus is likely the result of a lack of herbaceous plants and low levels of floral resources in these forests, which are key limiting factors for pollinators^28^. In terms of forest production, mixed-species plantations are known to have equal or higher productivity than monocultures^33^,^34^. The similarity in profit returns from and labour inputs to GFGP monocultures versus mixed forests (which are themselves mostly fine-scale checkerboards of monocultures) is unsurprising, given that the study region's small, sloped landholdings preclude the potentially lower production costs of monocultures that could arise from the economies of scale^35^. Finally, south-central Sichuan is undergoing a profound socioeconomic transformation wherein rural income is increasingly coming from urban wages or other non-farm professions^36^. Forestry on small landholdings is thus unlikely to be a prominent household income source now and in the coming years^37^.

The biodiversity gap between all GFGP forests and degraded native forest reveals the magnitude of potential biodiversity gains if GFGP aimed to restore native rather than structurally and compositionally simple forests in south-central Sichuan, although the recovery of biodiversity may still lag years behind forest establishment^31^. Moreover, insights from other ecosystems suggest that GFGP's primary goals of preventing soil erosion and fostering forest production could be fulfilled by native forests, as well as if not better than by monocultures and simple mixed forests^1^,^38^,^39^,^40^. We do not yet know the ecological or economic challenges of restoring native vegetation in this region. However, research in other ecosystems, including elsewhere in China^41^, points to promising outcomes of native forest restoration programs^42^,^43^. As noted earlier, our estimate of the opportunity cost of foregoing GFGP forest production was based on the assumption of zero profit from native forest; it is very likely an overestimate of the true cost given that native forest can generate a range of production and non-production incomes^1^,^30^. We believe a top research priority should be to determine the extent to which restoring native forests can deliver GFGP's goals in a sustainable and cost-effective way. Such research will help to determine whether native forest restoration can be made a component of future GFGP or other reforestation efforts in the region.

Within the current modes of GFGP reforestation, the promotion of mixed forests over monocultures in south-central Sichuan will not only bring modest biodiversity gains but may also generate other environmental and economic benefits^44^,^45^, including those highly valued under GFGP's mission hierarchy. For example, soil erosion control—GFGP's central mission—is probably better achieved by mixed forests than by monocultures^38^, especially monocultures with minimal ground cover^46^ such as bamboo and Japanese cedar. The generally equal or higher productivity of mixed forests over monocultures^33^,^34^ suggests equal or higher potential for carbon sequestration^47^,^48^. Compared with monocultures, mixed forests tend to be more resilient to pests^49^ and market price fluctuations of forest products^45^. To the best of our knowledge, no studies have directly evaluated multiple environmental and/or economic co-benefits of mixed forests versus monocultures under GFGP, or what forms of mixed forests (patch- versus tree-level mixture, different combinations of tree species and so on) most effectively deliver these benefits. Within the constraints of GFGP's current modes of reforestation, these topics should be given more research attention in order to increase GFGP's environmental and economic benefits.

Four caveats to our regional findings warrant discussion. First, limitations with respect to the selection of study sites may have biased our evaluation of the relative biodiversity impacts/potential of GFGP forests. Because of the extent of GFGP reforestation, in order to find suitable expanses of cropland to use as a baseline, we were forced to look to croplands on generally flat terrain, which are likely subject to more intense farming practices^15^,^23^ and, therefore, possibly support less biodiversity^24^,^25^ than did the sloped, low-intensity croplands that were reforested under GFGP. Alternatively, croplands on flat terrain could also be more fertile than sloped cropland, thus supporting more biodiversity through higher ecosystem productivity^26^. Our study may thus have over- or under-estimated the biodiversity impacts of GFGP reforestation of cropland. Similarly, our survey sites for native forest are mostly concentrated around Emei Mountain, which is a protected area, while survey sites for other land-cover types are generally not in the vicinity of protected areas. Source populations^50^ of birds and bees in Emei Mountain could thus have contributed to the higher biodiversity of native forests compared with GFGP forests, potentially exaggerating the conservation value of native-forest reforestation. However, considering the obvious differences in vegetative features between cropland/native forest and GFGP forests (Supplementary Note 1), we believe that these potential biases are unlikely to have driven our major findings^31^,^32^. Second, our inclusion of forest stands of different growth stages and management intensities likely increased the variation in biodiversity patterns associated with each forest land-cover type, because forest stand age and management intensity are known to influence biodiversity^31^. The fact that we included only closed-canopy forest stands reduces such variation to some extent and ensures that our findings apply to forest growth stages that prevail for most of the harvest cycles and the widest spatial coverage of each given type of GFGP forest. Moreover, the short rotation cycles for GFGP forests should alleviate to some extent the effects of differences in stand age on biodiversity patterns. However, such variation may still have affected the overall biodiversity contrast between GFGP forests and baseline land-cover types. Third, because of the spatial distribution of target land-cover types in the study region, our sampling sites tended to be clustered together within each land-cover type. Although we tried to maximize the spatial independence of sampling points/plots by placing them a minimum distance apart and by covering at least two clusters of survey expanses spaced ≥15 km apart, issues pertaining to spatial autocorrelation and site idiosyncrasies may still have biased our biodiversity inferences. Finally, our economic analysis is based on a relatively small sample size. Our economic conclusions on GFGP forest production should thus be taken with caution; assessments based on larger sample sizes would yield more robust conclusions.

A key question is whether the biodiversity and economic findings from our field study in south-central Sichuan hold elsewhere in China's GFGP forest landscapes. We cannot answer this question based solely on our work in one region. Nonetheless, while GFGP forests across China vary in species composition (Supplementary Data 1), they are all essentially monocultures or mosaics of a small number of species (Fig. 1). In this sense, by including three types of monocultures plus compositionally simple mixed forest, our field study covered the national spectrum of GFGP forest types. Our biodiversity findings thus stand to inform biodiversity patterns under GFGP in other regions of China with similar biophysical conditions^31^,^32^,^51^,^52^. In addition, the socioeconomic factors that underlie our economic findings, that is, small, sloped forest landholdings and an ongoing rural socioeconomic transition, apply to most areas where GFGP has been implemented^10^,^36^. Thus, we have reason to expect that our major biodiversity and economic findings will mirror trends elsewhere in China. Still, given the vastness and diversity of China, studies similar to ours from other regions will be critical to understanding the full biodiversity implications of GFGP, particularly in areas with different biophysical and socioeconomic conditions^53^.

We offer two policy recommendations pertaining to improving GFGP's biodiversity impacts in south-central Sichuan. First, the conservation and restoration of native forest will produce major benefits for the region's biodiversity and therefore warrant serious consideration by the government, possibly as an additional goal of GFGP. Second, within the constraints of current incentives and policies (and thus current modes of reforestation), GFGP can achieve better biodiversity outcomes at essentially no cost to households by promoting mixed forests over monocultures. Thus, as the current monocultures in south-central Sichuan are harvested, they should be replaced with mixed forests. Only additional research can reveal the degree to which these recommendations apply to other regions of China, and we highlight the urgency of such research. Given the vast scale of GFGP, this programme will play an enormous role in determining the future of biodiversity in China. More generally, as reforestation assumes an increasingly important role in meeting the environmental and livelihood challenges posed by deforestation^1^,^2^, we need to identify cost-effective opportunities for biodiversity conservation under different modes of reforestation, including the restoration of native forests.

## Methods

### The type and extent of GFGP forests across China

We extensively reviewed the peer-reviewed literature in both English and Chinese (including degree thesis; Supplementary Methods). To search the English literature on Web of Science ( www.webofknowledge.com), we checked all publications cited in or citing four ‘anchor' publications on GFGP that are foundational or comprehensive in scope^6^,^9^,^15^,^48^ and all publications specifically on biodiversity under GFGP (Supplementary Methods). To search the Chinese literature on the China Knowledge Resource Integrated Database ( www.cnki.net), we used the Mandarin term ‘tui geng huan lin' (‘
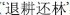
', the official name of GFGP in Chinese) as the only search term in the topic field. We retained those publications that provided information on the type of GFGP forests based on tree composition, and compiled location-specific information on GFGP forest types. Most studies did not specify the scale at which the type of GFGP forests was defined; it is therefore possible that some reported monoculture stands, if small and adjacent to stands of different tree species, may form mixed forests at larger spatial scales. We compiled all information before the start of our field study in November 2013; results were current as of 20 October 2015.

### Identifying field sites in south-central Sichuan

We used published geographic information on land-cover change (2000–2012 (ref. 54)) to identify major locations in our study region that underwent reforestation roughly within GFGP's time frame. We then visited these locations and conducted informal household interviews to confirm that there had indeed been extensive reforestation under GFGP in these locations and to identify the major forest types resulting from GFGP reforestation. For the purposes of bird and bee surveys (see below), we included only habitat expanses ≥60 ha in size to reduce edge effects. We adopted this minimum size based on a minimum length and width of 2 km and 300 m, respectively, for forest expanses occurring on mountain slopes. The narrow width of forest expanses is because in the study region, GFGP reforestation has taken place mostly on sloped terrain, predominantly on mountain slopes whose accessible sections are generally no more than 300 m wide. The designation of forest types was thus done on a minimum scale of 60 ha.

### Biodiversity surveys

We selected large expanses (≥60 ha) of each land-cover type, stratified into three elevation bands (Supplementary Table 2; Supplementary Methods), to conduct bird and bee abundance surveys; for each land-cover type, we used at least two clusters of survey expanses spaced ≥15 km apart to minimize potential idiosyncrasies related to the location of the survey expanses. We did not include scrubland (another land-cover type reforested under GFGP (ref. 8)) because it was virtually nonexistent in the region at the time of our study. While our biodiversity surveys of GFGP forests covered forest stands of different growth stages and management intensities, we excluded from biodiversity surveys forest stands that had not attained closed canopy; for eucalyptus and Japanese cedar, we thus surveyed only forest stands that were at least three and five years old, respectively. Bamboo stands encountered in our study always had closed canopy. Mixed forest stands predominantly consisted of bamboo plus Japanese cedar; we excluded mixed forest stands in which the Japanese cedar was <5-year-old (per our criterion for Japanese cedar monocultures).

We determined survey effort within land-cover types and elevation bands based on the saturation of species accumulation curves for birds. In the case of bees, the accumulation curves did not saturate; we therefore conducted trapping as much as our time and staffing allowed, and aimed to have a minimum of ten trapping plots for each land-cover type. For bird surveys, we conducted 12-min, 150 m-radius point counts spaced ≥250 m apart (avoiding double-counting) covering all species except aerial ones, and we accounted for imperfect detection using the time-of-detection approach^55^. For bird surveys during the non-breeding season, when a considerable proportion of the bird community exists in the form of mobile mixed-species foraging flocks, we additionally quantified the composition of flocks encountered during the time of point counts and/or of travel between point counts, and we combined data from point counts and flock observation to estimate population abundance (Supplementary Methods). For bee surveys, we delineated 1-ha (100 m × 100 m) trapping plots spaced ≥300 m apart, in each of which we operated 40 fluorescent pan traps for 24 h (ref. 56) (Supplementary Fig. 4; Supplementary Methods). All required permits for animal sampling were obtained from the IACUC (Institutional Animal Care and Use Committee) of Princeton University.

### DNA barcoding

We collected and individually stored all bees and bee-like insects (in case of visual misidentification) captured in pan traps in 2 ml tubes that were two-thirds filled with 99.99% ethanol. All samples were stored to −20 °C within five days of field collection until lab work. For each individual, we extracted DNA from one leg, and amplified and sequenced the 658-bp barcode region of the mtCOI gene^57^,^58^. After sequence quality control, pairwise alignment and a translation check, we used MOLE-BLAST ( www.blast.ncbi.nlm.nih.gov/moleblast/moleblast.cgi; accessed on 30 July 2015) to filter out non-bee sequences. For all 546 bee sequences, we applied single-threshold generalized mixed yule coalescent analysis (GMYC) to delimit species^59^. See Supplementary Methods for further details.

### Household interviews

In villages around our biodiversity survey sites, we randomly selected 166 households (≥35 households for each type of GFGP forest) with the constraints that (1) the household head was available for interviews and able to provide clear answers to our questions, (2) no more than three households were from the same village and (3) households from a given village covered a spectrum of landholding size and economic status. We interviewed household heads for 30–40 min using a questionnaire that we had previously piloted and tailored for use in the study region. Most importantly, the questionnaire asked respondents to self-report the percentage of household income contributed by forest production, and broke down the costs of and income streams from forest production (for example, seedling, fertilizer and/or labour cost during forest establishment, maintenance, and selective/clear-cut harvest and so on; Supplementary Methods). We conducted all interviews in July 2015. All required permits for household interviews were obtained from the IRB (Institutional Review Board) of Princeton University, and all respondents gave informed consent before the interviews.

### Statistical analysis

Unless noted otherwise, we used *R* 3.1.3 (ref. 60) for all analyses. Considering the likely inadequate sampling of bees, we omitted five outlier plots (two for cropland and bamboo monoculture each, one for native forest) because of their potentially large influence on data patterns, based on the total number of individuals trapped (Supplementary Methods). For biodiversity analyses at the community level, we compared species richness and community composition between each type of GFGP forest and the baseline land-cover types of cropland and native forest. At the species level, we compared species abundance in each type of GFGP forest with the baseline land-cover types, and tallied the number of species associated with only one land-cover type; the latter analysis of ‘habitat specialists' provides an assessment of how different land-cover types provide unique habitat for biodiversity at the regional landscape scale.

We estimated comparable species richness in each land-cover type using coverage-based extrapolation^61^,^62^ for birds (package *iNEXT* version 2.0.1 (ref. 63)) and sample-based extrapolation^62^,^64^ for bees (programme *EstimateS* version 9.1.0 (ref. 65)). Both extrapolation methods correct for incomplete species detection and thus allow comparisons of species richness among communities^62^; coverage-based extrapolation standardizes on sample completeness (that is, coverage)^61^, while sample-based extrapolation standardizes on sampling effort (that is, the number of sampling units)^64^. We used sample-based extrapolation for bees because our less adequate sampling effort for bees resulted in very low coverage, potentially rendering coverage-based richness comparisons less reliable and less meaningful^62^ (see Supplementary Table 8 for the extrapolated coverage/sample size). We tested for community compositional turnover between GFGP forests and baseline land-cover types using PERMANOVA (refs 66, 67; Supplementary Methods) and tallied the number of species not shared between communities. We classified bird species into three guilds of habitat association (forest-dependent, generalist and open-country species^68^,^69^; Supplementary Data 6), and tallied the number of species belonging to each guild in each community. At the species level, we compared the abundance of each species among land-cover types using N-mixture models^70^ for birds, and generalized linear models for bees because the limited capture of bees made it challenging to account for capture rate. We applied this analysis to species with ≥10 total detections/captures and to certain bird genera into which we collapsed constituent species when only the genus satisfied the 10-detection/capture requirement (Supplementary Methods). Because we analysed multiple species, we corrected *P* values for N-mixture models/generalized linear models using the Benjamini–Hochberg correction for multiple testing, which combines information on the original *P* values, the total number of tests and the false discovery rate (that is, the proportion of false positives considered allowable) to calculate corrected *P* values^29^; we used a false discovery rate of 0.1 (that is, we were willing to accept up to 10% of the significant results being false positives).

For household interview data, we compared the percentage of household income contributed by forest production, forest profit and labour intensity among different types of GFGP forests using multiple linear models, after accounting for cost and benefit discount over time (Supplementary Methods). For the percentage of household income contributed by forest production, we eliminated four outlier data points of excessively high percentage (two for bamboo monoculture and two for mixed forest); for the calculation of forest profit and labour intensity, we removed estimates for which one or more components of the cost/income appeared unrealistic (Supplementary Methods). From the 166 respondent households, we thus obtained 105 estimates for self-reported household income percentage, 54 for forest profit and 112 for labour intensity (Supplementary Table 4). We applied a discount rate of 5% (*r*=0.05) to production sale, cost and labour input based on the 2015 1-year lending rate of the People's Bank of China (range 4.35–6%^71^). Alternative discount rates (*r*=0, 0.0125 and 0.025; 1-year interest rate for personal saving was 1.35–1.75% as of 24 October 2015 (ref. 71)) did not qualitatively change the conclusions (Supplementary Table 6).

### Data availability

The authors declare that the literature review data supporting the findings of this study are available as Supplementary Data 1. The biodiversity and economics field data that support the findings of this study are available in the Dryad Digital Repository with the identifiers doi:10.5061/dryad.14c6b (ref. 72).

## Additional information

**How to cite this article:** Hua, F. *et al*. Opportunities for biodiversity gains under the world's largest reforestation programme. *Nat. Commun.* 7:12717 doi: 10.1038/ncomms12717 (2016).

## Supplementary Material

Supplementary Information Supplementary Figures 1 - 4, Supplementary Tables 1 - 8, Supplementary Note 1, Supplementary Methods and Supplementary References

Supplementary Data 1 Records of GFGP forests and their vegetation make-up reported in field-based studies.

Supplementary Data 2 Species' detection status (detection represented by "≥") in each land-cover type.

Supplementary Data 3 Abundance difference of species/genera between GFGP forests and cropland for avian breeding and nonbreeding seasons.

Supplementary Data 4 Abundance difference of species/genera between GFGP forests and native forest for avian breeding and nonbreeding seasons.

Supplementary Data 5 List of habitat specialists.

Supplementary Data 6 Assignment of bird species into habitat guilds.
