# Supplementary material for: Opportunities for biodiversity gains under the world's largest reforestation programme
Source: Nat Commun. 2016 Sep 6;7:12717. doi: 10.1038/ncomms12717 (PMC5025860; doi:10.1038/ncomms12717)
Supplement: Supplementary Information — Supplementary Figures 1 - 4, Supplementary Tables 1 - 8, Supplementary Note 1, Supplementary Methods and Supplementary References [file ncomms12717-s1.pdf]

Supplementary Figures

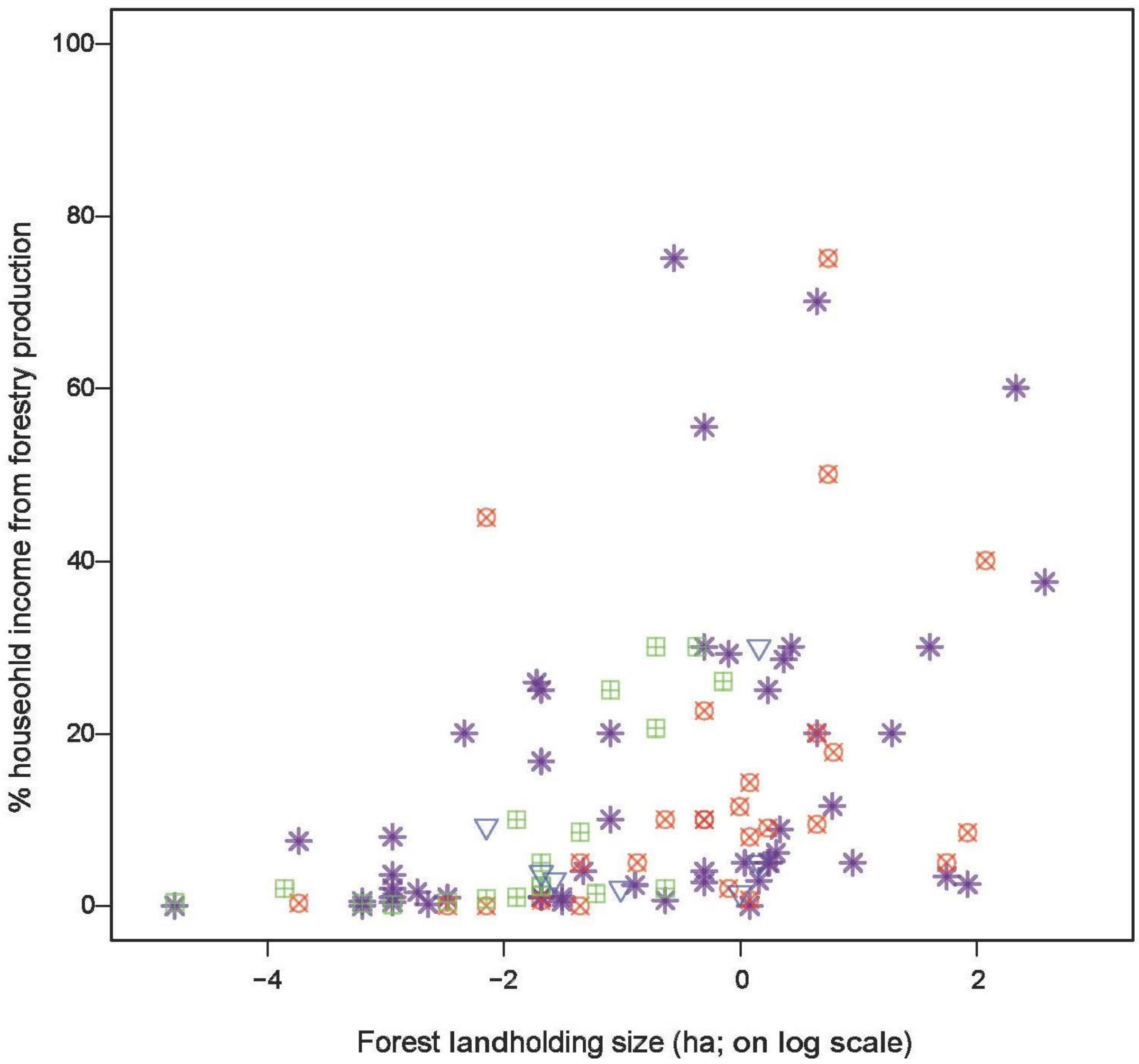

Supplementary Figure 1. Distribution of respondent households in terms of the percentage of household income from forest production against forest landholding size. Symbols of different shapes and colors represent households managing different types of forest: green crossed square - eucalyptus monoculture, blue triangle - bamboo monoculture, red crossed circle - Japanese cedar monoculture, and purple asterisk - mixed forest. Forest landholding size is displayed on a log scale. The percentage of household income from forest production is positively correlated with forest landholding size (β = 4.20, SE = 1.17, 95% confidence interval = [1.91, 6.49]).

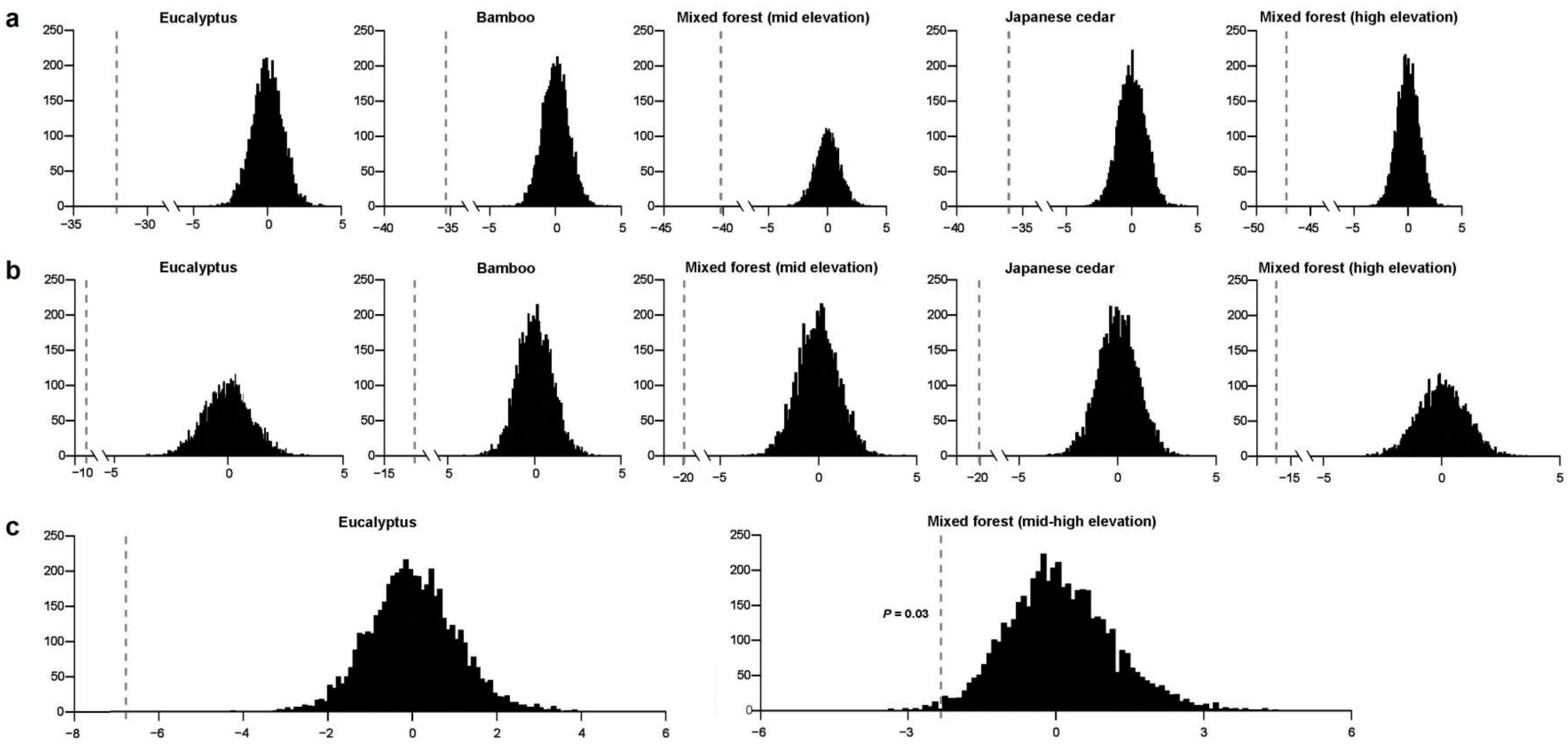

Supplementary Figure 2. Community compositional differences between GFGP forests and cropland based on PERMANOVA analysis of community dissimilarity under 5000 permutations. Graphs are 100-bin histograms (black) with X axis displaying the t-test statistic score that compares (1) community dissimilarity (using Bray-Curtis dissimilarity index) within the GFGP forest type in question against (2) community dissimilarity between the GFGP forest type in question and cropland, and Y axis displaying the number of permutations yielding each bin of t-test statistic score; grey dotted lines display the observed t-test statistic score. Panels display analyses for birds during the breeding (a) and nonbreeding seasons (b), and bees (c). All PERMANOVA tests where grey dotted line does not overlap with black histogram have $P < 0.001$; P-values are displayed for tests where $P \geq 0.001$.

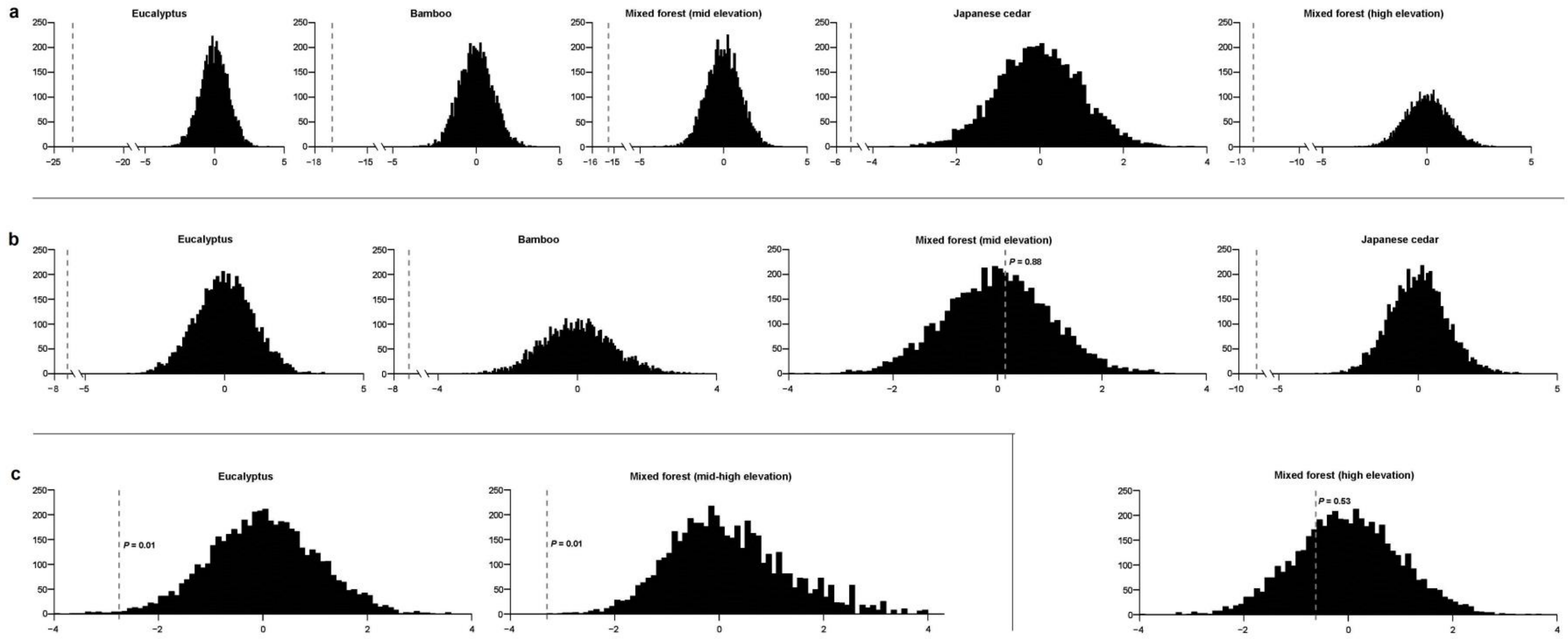

Supplementary Figure 3. Community compositional differences between GFGP forests and native forest based on PERMANOVA analysis of community dissimilarity under 5000 permutations. Graphs are 100-bin histograms (black) with X axis displaying the t-test statistic score that compares (1) community dissimilarity (using Bray-Curtis dissimilarity index) within the GFGP forest type in question against (2) community dissimilarity between the GFGP forest type in question and native forest, and Y axis displaying the number of permutations yielding each bin of t-test statistic score; grey dotted lines display the observed t-test statistic score. Panels display analyses for birds during the breeding (a) and nonbreeding seasons (b), and bees (c). All PERMANOVA tests where grey dotted line does not overlap with black histogram have $P < 0.001$; P-values are displayed for tests where $P \geq 0.001$.

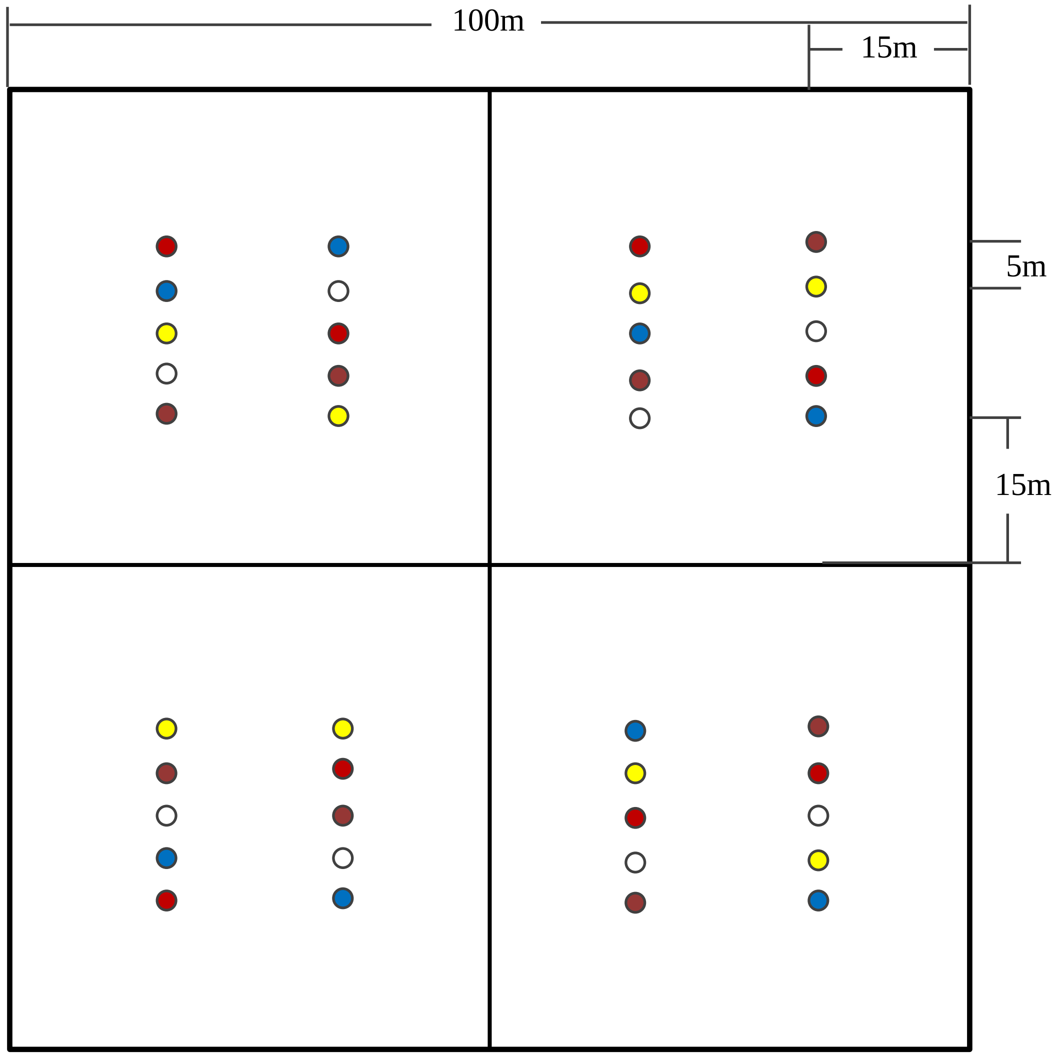

Supplementary Figure 4. Schematic illustration of pan trap layout in a trapping plot. Color dots represent individual pan traps of different colors (dyed with fluorescent spray paint).

Supplementary Tables

Supplementary Table 1. Information synopsis on six target land-cover types included in field study in south-central Sichuan Province.

Land-cover category	Land-cover type	Forest structure/main crop	Elevation	Note on condition and production mode
GFGP forest	Eucalyptus	Broadleaf	≤ 650 m (low)	• Monoculture; for timber production.
	Bamboo†	Bamboo	530-1000 m (mid)	• Monoculture; for timber and bamboo shoot production.
	Japanese cedar	Coniferous	≥ 830 m (high)	• Monoculture; for timber production.
	Mixed forest	Mixed broadleaf and coniferous	≥ 530 m (mid & high)	• Compositionally simple, involving 2-5 tree species and predominantly a bi-mixture of bamboo and Japanese cedar; for timber and bamboo shoot production.
Baseline land cover	Cropland	Seasonally rotational rice, corn, vegetables	All elevations	• Non-mechanized, generally low-intensity farming; proxy for GFGP-replaced cropland.
	Native forest	Broadleaf	All elevations	• Disturbed, often with regular extraction of (non)timber forest products; proxy for the most conservation-friendly forest form conforming to the production, unprotected nature of GFGP forests.

Note: † - In this study, bamboo stands involving multiple bamboo species were considered as bamboo monoculture because of the similar and consistently simple forest structure associated with all bamboo species concerned in this study.

Supplementary Table 2. Thresholds of elevation for assigning elevation bands and the number of sampling units shared between neighboring elevation bands.

Field season	Elevation band	Elevation bound (m)		Number of survey units shared with the next higher neighboring band		
		Lower	Upper	Land cover	Bird point count/mixed-species flock	Bee trapping plots
Avian breeding	Low	342	647	Cropland	17	0
				Native forest	13	4
	Mid	529	1017	Cropland	14	--
				Native forest	9	--
				Mixed forest	12	--
	High	878	1429	Cropland	--	--
				Native forest	--	--
				Mixed forest	--	--
Avian nonbreeding	Low	315	648	Cropland	11/2	--
				Native forest	27/7	--
	Mid	532	1102	Cropland	32/1	--
				Native forest	37/7	--
				Mixed forest	26/4	--
	High	829	1314	Cropland	--	--
				Native forest	--	--
				Mixed forest	--	--

Supplementary Table 3. Tally of bird and bee survey sample size.

Taxon	Elevation band	Land-cover type	Sample size†	
			Avian breeding season	Avian nonbreeding season§
Bird	Low	Cropland	53	48/3
		Native forest	35	28/7
		Eucalyptus forest	57	54/5
	Mid	Cropland	46	43/3
		Native forest	46	80/19
		Bamboo forest	55	49/4
		Mixed forest	66	59/8
	High	Cropland	46	49/1
		Native forest	67	77/23
		Japanese cedar forest	72	57/8
		Mixed forest	84	65/5
Bee	Low	Cropland	6‡	--
		Native forest	5	--
		Eucalyptus forest	10	--
	Mid-high	Cropland	9	--
		Native forest	15‡	--
		Bamboo forest	8‡	--
		Japanese cedar forest	12	--
		Mixed forest	10	--

Note: † - Sample size is in terms of the numbers of point counts (birds), trapping plots (bees) and mixed-species flocks recorded (birds), by land-cover type and season. ‡ - Based on Tietjen-Moore test ($P < 0.05$), we removed two cropland plots in low elevation, as well as one native forest plot and two bamboo plots in mid-high elevation as outliers. § - Numbers before and after the slash refer to the numbers of point counts and mixed-species flocks observed, respectively.

Supplementary Table 4. Tally of household interview sample size by GFGP forest type.

GFGP forest type	Sample size (number of households)		
	Percentage of household income from forest production	Production profit	Labor intensity
Eucalyptus	20	20	14
Bamboo	7	12	14
Japanese cedar	26	10	43
Mixed forest	52	12	41
Total	105	54	112

Note: Household interview sample size is in terms of the numbers of estimates obtained for production profit, labor intensity, and percentage of household income coming from forest production.

Supplementary Table 5. The number of species shared between each type of GFGP forest and baseline land cover that had different abundances[†] between each type of GFGP forest and baseline land cover.

Taxon	Season	Type of GFGP forest	Baseline land cover					
			Cropland			Native forest		
			Higher	Lower	No difference	Higher	Lower	No difference
Bird	Breeding	Eucalyptus	2	1	29	0	0	31
		Bamboo	5	6	21	3	4	25
		Mixed forest – mid elevation	7	8	20	4	2	28
		Japanese cedar	5	3	25	2	8	29
		Mixed forest – high elevation	7	3	23	2	7	31
	Nonbreeding	Eucalyptus	0	2	22	0	2	18
		Bamboo	0	0	16	0	1	18
		Mixed forest – mid elevation	0	0	23	0	0	30
		Japanese cedar	0	0	19	0	3	20
		Mixed forest – high elevation	0	0	22	2	5	23
Bee	NA	Eucalyptus	0	1	2	1	1	1
		Bamboo	0	1	2	0	1	0
		Japanese cedar	0	1	2	0	0	1
		Mixed forest	0	2	1	0	1	1

Note: † - For a given species/genus, the abundance analysis compares its abundance in each type of GFGP forest against that in each type of baseline land cover. “Lower” and “higher” abundances therefore refer to lower and higher abundance in GFGP forest than in baseline land cover, respectively.

Supplementary Table 6. Comparison of forest annual profit (US\$ ha^{-1} yr^{-1} and labor intensity (Days ha^{-1} yr^{-1}) under different discount rates.

Discount rate	Comparison in	Forest type	β	SE	*P* (difference from mixed forest)
0.05	Forest profit	Bamboo	425.56	82.65	0.586
		Eucalyptus	497.71	70.78	0.211
		Japanese cedar	288.54	91.37	0.542
		Mixed forest	362.77	79.13	--
	Labor intensity	Bamboo	5.73	4.19	0.062
		Eucalyptus	13.70	3.54	0.832
		Japanese cedar	9.55	2.34	0.111
		Mixed forest	14.58	2.07	--
0	Forest profit	Bamboo	695.70	135.40	0.812
		Eucalyptus	726.10	116.00	0.934
		Japanese cedar	607.70	149.70	0.506
		Mixed forest	740.60	129.70	--
	Labor intensity	Bamboo	8.39	6.41	0.068
		Eucalyptus	15.27	5.42	0.315
		Japanese cedar	14.33	3.59	0.131
		Mixed forest	21.61	3.17	--
0.0125	Forest profit	Bamboo	610.39	117.10	0.985
		Eucalyptus	660.52	100.30	0.755
		Japanese cedar	499.18	129.40	0.508

		Mixed forest	613.37	112.09	--
	Labor intensity	Bamboo	7.56	5.71	0.066
		Eucalyptus	14.84	4.83	0.416
		Japanese cedar	12.82	3.19	0.126
		Mixed forest	19.40	2.82	--
0.025	Forest profit	Bamboo	538.46	102.83	0.850
		Eucalyptus	601.01	88.06	0.501
		Japanese cedar	412.92	113.68	0.516
		Mixed forest	511.44	98.45	--
	Labor intensity	Bamboo	6.85	5.12	0.065
		Eucalyptus	14.44	4.33	0.539
		Japanese cedar	11.55	2.86	0.121
		Mixed forest	17.53	2.53	--

Supplementary Table 7. Results of multiple linear model analysis of the percentage of household income coming from forest production.

Predictor variable	Tree species concerned	β	SE	95% CI
Forest type	Eucalyptus	7.72	3.69	[0.49, 14.95]
	Bamboo	5.52	6.09	[-6.42, 17.46]
	Japanese cedar	9.48	3.45	[2.72, 16.24]
	Mixed forest	9.67	2.58	[4.61, 14.73]
Area of forest holding	--	4.20	1.17	[1.91, 6.49]

Supplementary Table 8. The coverage (birds†) and sample size (bees‡) sampling data were extrapolated to in the analysis of species richness.

Taxon	Low elevation	Mid elevation	High elevation
Birds (breeding)	98.8%	97.9%	98.8%
Birds (nonbreeding)	98.4%	99.4%	99.3%
Bees	10	--	16 (Mid-high elevation)

Note: † - Coverage is a measure of the completeness with which sampling data represent the species richness of a community; specifically, it is defined as the percentage of the total number of individuals in a community that belong to species represented in the sample [1]. ‡ - Sample size refers to the number of trapping plots.

Supplementary Notes

Supplementary Note 1. Details on the vegetative make-up of the six target land-cover types

Eucalyptus forest. The tree species used in eucalyptus monoculture forests is *Eucalyptus grandis* W. Hill ex Maiden, which is not native to China. Individual trees are typically spaced 2 m apart, and harvested using clear-cuts every six to seven years. Forest stands are typically managed with chemical weeding and fertilization, which results in a highly limited understory structure, although on the rare occasions that forest stands are subject to less management, they usually have a well-developed understory.

Bamboo forest. Tree species used in bamboo monoculture forests mostly include *Neosinocalamus affinis* (Rendle) Keng f, *Pleioblastus amarus* (Keng) Keng, and *Phyllostachys pubescens* Mazel ex H. de Lehaie. All species are native to the region. Spacing between individual plants varies depending on the species. Typically, *Neosinocalamus affinis* and *Phyllostachys pubescens* bamboo forests are selectively harvested every one to two years for timber, while *Pleioblastus amarus* forests are maintained for bamboo shoot production. Forest stands are typically managed with chemical fertilization; minimal weeding is needed because the strong water-absorbing ability of bamboo plants discourages the growth of other plants. In all cases, understory structure is minimal.

Japanese cedar forest. The tree species used in Japanese cedar monoculture forests is *Cryptomeria japonica* var. *sinensis* Miquel, which is native to the region. Young plantations are started at high densities, and trees are then selectively removed until a harvest density of two to three meters apart. The mode of selective harvest (i.e., timing and number of harvests) tends to vary among households. Forest stands are typically managed with chemical fertilization. Weeding is only done before canopy closure, after which the stands self-maintain minimal

understory structure because of extremely low light levels in the understory. Clear-cut typically happens at 18-20 years.

Mixed forest. Mixed GFGP forests consist of up to five, although most typically two or three tree species, including alder (*Alnus cremastogyne* Burk), Japanese cedar (*Cryptomeria japonica* var. *sinensis* Miquel), bamboo (*Neosinocalamus affinis, Pleioblastus amarus*, *Phyllostachys pubescens*), toona (*Toona ciliata* Roem), and happy tree (*Camptotheca acuminata* Decne). All species are native to the region. Stands can follow one of two levels of species mixture: mixture of individual trees/plants (hereafter "tree-level mixture"), or mixture at the scale of small patches of monocultures (hereafter "patch-level mixture"). Plots consisting of a patch-level mixture tend to be managed in the same way as monoculture forest described above. Spacing between individual trees varies between species. For tree-level mixtures, forest stands are sometimes managed with chemical weeding and fertilization. Stands are typically selectively harvested on cycles specific to different species. Understory structure is typically reasonably developed for forests with tree-/plant-level mixture, and understory structure of patch-level mixture generally resembles that of the corresponding monoculture plots.

Cropland. Croplands included in our field study are invariably distributed ≤ 5 km from the surveyed forest expanses and on relatively flat terrain, and are predominantly managed in a low-intensity way that involves minimal mechanization. Crops at the time of the avian breeding season surveys were predominantly rice, corn, and vegetables; crops at the time of avian nonbreeding season surveys were predominantly fallow rice paddies (no crop) and vegetables.

Native forest. Native forest in the region is mostly broadleaf subtropical evergreen forest, dominated by tree species in the families Lauraceae, Fagaceae, Theaceae and Symplocaceae, with well-developed herbaceous understory. Native forests sites in our field study consisted of

sites in and around the Emei Mountain Forest Park (> 98% of survey efforts) and the Xiang'er Buddhist temple in Pengshan County of Meishan Municipality (Fig. 2). The vast majority of forests surveyed were subject to considerable levels of disturbance and resource extraction including timber and herbal medicine collection. Notably, forests outside of Emei Mountain Forest Park are routinely dotted with small, scattered stands of monoculture plantations that have replaced former native forest cover.

Supplementary Methods

Compilation of peer-reviewed literature on GFGP forest type. To search the English literature on Web of Science (www.webofknowledge.com), we checked all publications cited in and citing four "anchor" publications on GFGP: two generic review articles [2,3], one meta-analysis on soil carbon levels [4], and one generic book [5]. We additionally searched for publications about biodiversity under GFGP, using the search terms "China", "grain for green", and "biodiversity". Importantly, we used all six variants of GFGP's English translation that have occurred in the literature, including "grain for green", "grain-for-green", "grain to green" (e.g. [2]), "grain-to-green", "sloping land conversion" (e.g. [6]), and "returning farmland to forest program" (e.g. [7]). For our search of the Mandarin literature on the China Knowledge Resource Integrated Database (www.cnki.net), we used the Mandarin term "tui geng huan lin" ("退耕还林", the official name of GFGP in Mandarin) as the only search term. Of all publications we retrieved, we retained only those that provided information on the type of GFGP forests (i.e. composition of tree species, or at least if the forest was monoculture or mixed forest).

Biodiversity sampling. All bird point count stations and bee trapping plots were ≥ 50 m from the edge of the focal land-cover type. We conducted all surveys at lower elevations before moving to higher elevations with the exception of a small subset of breeding season point counts and ~25% of the bee trapping plots at low elevations during the avian breeding season, which we surveyed toward the latter half of the field season. We determined the required effort level of point count surveys for each land-cover type based on the leveling of species accumulation curves. Pan trapping efforts were limited by time and personnel; we had a minimum of ten trapping plots for each land-cover type. For both birds and bees, survey effort was higher for the more biodiverse land-cover types (according to our field experience).

For bird point count surveys, we used a 150-m radius, avoiding double-counts to the best of our ability. We divided each 12-minute survey into four 3-min subintervals and recorded individual-/group-level detections (using visual and auditory cues) for each subinterval [8], excluding flying individuals. In the nonbreeding season, when there were many mixed-species flocks, we additionally quantified the composition of flocks encountered during the point counts and/or during travel between point counts. To characterize mixed-species flocks during the nonbreeding season, we recorded the species identity and number of all individuals in the flocks that we encountered. Observers were allowed to move up to 10 m from the routes between point count stations, which we considered a realistic distance for the auditory detection of passing flocks, and to spend up to five minutes observing each flock. In situations where a flock passed through during a point count period, we stopped the point count and instead focused on recording the flock, before restarting the point count from the beginning. Breeding season surveys were conducted between 30 minutes before dawn and 10:30 am, and nonbreeding season surveys between 8:30 am and 4:30 pm. Two different groups consisting of four surveyors each conducted the breeding and nonbreeding season surveys. We minimized observer bias with regard to land-cover type by ensuring that each land-cover type was covered by all observers, and that observer identity was considered in subsequent analysis (see "Statistical analysis" below). Supplementary Table 3 lists the number of point counts conducted for each land-cover type and season, divided into elevation bands (see elevation band delineation below and in Supplementary Table 2).

For bee surveys, we divided each trapping plot into four equal quadrats, within each of which we systematically set up two parallel lines of five fluorescent pan traps of different colors spaced 5 m apart and 15 m from quadrat edges; each trapping plot thus had 40 traps (Supplementary Fig. 4). We used 96 ml plastic pans (3.25-ounce translucent plastic soufflé

portion cups; www.cuptainers.com) that we fluorescent-dyed with white, red, yellow, blue and purple, respectively. We set up all pans on 1-m poles to improve visibility, filled each pan two thirds full with 2% scentless liquid detergent as a surfactant [9] , and operated them for 24 hours on days without rain. We collected all captured individuals (including bees and other insects) and stored them in 99.99% ethanol at ≤ 4°C before DNA extraction. All samples were stored at -20 °C within five days of field collection until the time of lab work. Seven out of the total 74 plots were changed to a rectangular shape of equivalent area due to terrain constraints. Supplementary Table 3 lists the number of trapping plots surveyed for each land-cover type, divided into elevation bands (see elevation band delineation below and in Supplementary Table 2).

DNA barcoding. For each individual, we extracted DNA from one leg following protocols in [10], and amplified the mtCOI gene using the corbiculate bee primers BarbeeF (5′-CAACAAATCATAAAAATATTGG-3′) and MtD9 (5′-CCCGGTAAAATTAAAATATAAACTTC-3′ [11]). Our PCR used 13.5ul reaction volumes that contained 1.25ul 10x buffer (Mg^{2+} plus), 0.2mM dNTPs, 0.25uM of each primer, 0.3U Taq DNA polymerase (TaKaRa Biotechnology (Dalian) Co., Ltd.), and ~60ng pooled genomic DNA. We implemented PCR amplifications with Mastercycler Pro (Eppendorf, Germany), starting with an initial denaturation process at 94 °C for 5 min, followed by 35 cycles, each comprising 94 °C for 1 min, 38 °C for 80s and 64 °C for 2 min, and finished with 64 °C for 10 min. We used the ABI 3730xl DNA Analyser (Applied Biosystems, Carlsbad, California, USA) for sequencing; after sequence quality control, pairwise alignment and a translation check, we obtained 734 Sanger sequences. We then used MOLE-BLAST (www.blast.ncbi.nlm.nih.gov/moleblast/moleblast.cgi; accessed July 30th 2015) to filter out non-bee sequences, and obtained 546 bee sequences.

For all mtCOI Sanger sequences, we conducted single-threshold GMYC species delineation [12]. We constructed an ultrametric gene tree under a relaxed log-normal molecular clock with BEAST 2.3.0, generated the BEAST input file with BEAUti 2.3.0 [13] and used the GTR substitution model, which we selected using jModelTest2 2.1.7 [14,15]. We set mean substitution rate to one, and estimated the proportion invariant and base frequencies. We chose a birth-death model as a single coalescent cluster constituting the GMYC null model. We set the AG transition rate prior to a gamma distribution with Alpha = 2 and Beta = 0.5 and all the other transition-rate parameters to gamma distribution with Alpha = 2 and Beta = 0.25. We set the ucldMean prior to 0.0176, which is the mean substitution rate for arthropods. We defined three Crabronidae COI sequences as the outgroup. We ran the MCMC chains for 20 million generations and sampled every 1000 generations. We visualized run convergence using Tracer version 1.6.0 [16], and discarded the first 10% of the trees as burn-in. We ran TreeAnnotator 1.8.2 [16] to produce a single tree for GMYC analysis using maximum clade credibility tree, with the Node Heights option set to Keep Target Heights. We applied single-threshold GMYC models to DNA barcodes using the package *splits* (1.0-19 [17]; in *R* 3.2.0 [18]; see enclosed R script: bees_GMYC_BEAST2.R).

Household interview. We obtained ≥50 interviews for each GFGP forest type, except for eucalyptus monoculture, for which we obtained 30 interviews (total number of households included was 166). Supplementary Table 4 lists the number of households interviewed for each land-cover type whose data contributed to our economic analyses. In each household, we first asked what forest types the household managed. For each forest type, we then asked about the management/production costs per unit area per production cycle, breaking the costs down into discrete processes, including the establishment, maintenance and harvesting of forests, and to

discrete components including costs of seedlings, chemicals, hired and self-labor, equipment and transportation. We also asked respondents about the yield and market price for forest products per unit area per production cycle, covering both timber and non-timber products. These questions allowed us to calculate the yield and profit of different GFGP forest types. Importantly, because of China's registration system that ties parcels of rural land to particular households, respondents generally know the sizes of their land-holdings with considerable accuracy. While government subsidies on seedlings and fertilizer could result in underestimations of production costs, there is no reason to expect this underestimation to systematically vary with GFGP forest type in a way that would bias our conclusions on profit from forest products. We additionally asked respondents to estimate what percentage of their household income came from forest production.

Statistical analysis. Considering the likely inadequate sampling of bees, we first eliminated plots identified as outliers of anomalous trapping patterns. To identify outlier plots, we tallied the total number of individuals trapped from each survey plot, and used the Tietjen-Moore test for outliers [19] to identify plots with excessively low or high number of captures ($P < 0.05$). This resulted in the removal of five survey plots (Supplementary Table 3). We stratified biodiversity analysis into three elevation categories using the natural elevation range of the three monoculture forests (eucalyptus, bamboo, and Japanese cedar; Supplementary Note 1): eucalyptus defining low elevation, bamboo mid elevation, and Japanese cedar high elevation. For the analysis of bees, we combined data for mid and high elevations because of limited sample size. We assigned survey data for each monoculture to their respective elevation bands, and survey data for cropland, native forest and mixed GFGP forest, three land-cover types that spanned more than one elevation bands, to elevation bands according to the threshold elevation values provided in

Supplementary Table 2; a portion of these data was used in more than one elevation bands because of the overlap between bands (Supplementary Table 2).

For species richness analysis using coverage-/sample-based extrapolation, we extrapolated to two times the minimum sample size or the largest sample size, whichever was greater [20] (Supplementary Table 8). We used PERMANOVA to test community compositional turnover between each type of GFGP forest and baseline land-cover types, by permutating the site-level (with a site being a point count station for birds, and a trapping plot for bees) Bray-Curtis distance of community composition 5000 times [21,22]. For bees, no PERMANOVA was conducted for bamboo and Japanese cedar GFGP forests because both forest types involved only two plots that yielded at least one bee individual, thus involving only one community dissimilarity measure. We followed del Hoyo et al. [23] and MacKinnon et al. [24] in classifying bird species into forest-dependent, generalist, and open-country guilds. A species was classified as forest-dependent if it is predominantly associated with forested habitat, i.e. its association with forested habitat was cited by the sources as being stronger than “occasional” or “sometimes”. Similarly, a species was classified as open-country if its association with open habitat was cited by sources as being stronger than “occasional” or “sometimes”. We classified the remaining species in between these two association categories into the generalist guild (Supplementary Data 6).

For N-mixture modeling of bird species abundance, we treated only species with ≥ 10 total detections of individuals or groups as standalone species, and collapsed the remaining species into their respective genera only when the genus satisfied the 10-detection requirement. Abundance modeling for species living in groups estimated the abundance of groups rather than individuals. The rationale for using this 10-detection requirement lay in the fact that N-mixture

models entail modeling both the “true” abundance and the detection probability of each species, involving a maximum of ten covariates in the most complete models (i.e. global models; see below). The convergence of these N-mixture models, particularly when involving a large number of covariates, depends upon a reasonable number of detections to provide sufficient information to tease apart the contribution of true abundance and detection to the observed abundance of the species. Because we are unaware of well-established guidelines for “a reasonable number of detections”, we chose a minimum of ten detections; it was found to work well for the purposes of our models.

For all species/genera, the model’s abundance component included land-cover type as the only candidate covariate and used a log link and Poisson error distribution. The detection probability component included land-cover type (in binary form of forest versus non-forest), observer identity and day of year (we considered May 3rd and December 1st as day 1 for avian breeding and nonbreeding seasons, respectively) as candidate covariates, and time as a nuisance covariate, and used a log link and binomial error distribution. Models for the nonbreeding season were simultaneously parameterized with data from mixed-species flocks, because flocks accounted for a considerable proportion of the bird individuals detected during the nonbreeding season (see below). We assumed closed population and constant detection probability during each point count [8]. For each species/taxon, we generated a full set of sub-models from the global model (i.e. using all combinations of candidate covariates), and ranked these models based on the AIC (Akaike’s information criterion [25]) score. For the model with the lowest AIC score, we checked its estimates to gauge whether they were generally consistent with our biological knowledge of the species/genus. If they were, we would select the model with the lowest AIC score as the best model; otherwise, we would move to the model with the next lowest AIC score

and conduct such check again, until we identified the model with the lowest AIC score that produced estimates consistent with our biological knowledge, as the best model. The reason for including our biological knowledge of the species/genus was that in some situations, the particular data structure could cause the models to produce unrealistic abundance and detection probability estimates even if they converged (e.g. exceedingly high densities of populations). Our biological knowledge of the species'/genera's likely abundance from our field experiences therefore serves as a safeguard against such unrealistic estimates. For GLMs of bee species abundance, we followed the same 10-capture criterion in identifying standalone bee species, and collapsed the rest into one taxon. All GLM models included land-cover type as the only covariate and used a log link and Poisson error distribution.

For the parameterization of N-mixture models for the nonbreeding season, we combined data from both point count and mixed-species flock observation (see "Biodiversity sampling" above for methods of flock observation). The combination of these two datasets to inform N-mixture models was based on two assumptions. (1) For a given species/genus, the same population density underlay the pattern of the species'/genus' detection during both point count and mixed-species flock observation; in other words, the numbers of individuals detected during point count and flock observation were the representation of the same underlying density of the species/genus. (2) Unlike during point counts, bird individuals in flocks had a perfect detection probability; in other words, we considered the identity and number of bird individuals recorded in mixed-species flocks to be an adequate representation of the composition of the flocks. We consider this assumption to largely hold because of the high activity levels and visibility of flocks. As with the breeding season, N-mixture modeling for species living in groups estimated

the abundance of groups rather than individuals; for this purpose, flock observation of these group-living species recorded the number of groups rather than individuals.

Given these two assumptions, for a given species/genus, the expected number of individuals/groups detected during point count and flock observation could be expressed mathematically based on parameters of population density, land area surveyed, and, for point counts, detection probability. This expression involves (1) multiplying population density with land area surveyed to express the "super" population associated with the land-cover type [26], (2) conducting Poisson draws from this super population to express the number of individuals/groups that were present, and thus available, to be detected by point count or encountered by flock observation, and for point count, (3) conducting binomial draws using detection probability to express the number of individuals/groups detected during point count. Parameters pertaining to population density and detection probability were estimated by N-mixture models, while those pertaining to the land area surveyed were known (see below). N-mixture modeling thus hinged on using the maximum likelihood estimate approach to estimate the unknown parameters by fitting the mathematical expressions of the expected number of detections to the observed data.

For each point count, the land area surveyed (represented as A_{pc}) was calculated as the area of the circle covered by the 150 m radius; it is thus:

$$A_{\text{point count}} = \pi \text{ x } 150 \text{ m}^2$$
$$= 7.07 \text{ x } 10^4 \text{ m}^2$$

For mixed-species flock observation, because the observation was not conducted at clear-cut sampling units, we considered an overall rectangular area covered by the total effort of flock observation for each land-cover type, as an approximation of the area surveyed for flocks within

the land-cover type in question. Correspondingly, we combined flock data within each land-cover type to tally the total number of individuals/groups detected in flocks for a given species/genus. The length of this rectangular area (represented as L) was determined by (1) the total number of point count stations for the land-cover type in question (represented as n, which was known; Supplementary Table 3), (2) the typical inter-station distance of 250 m, and (3) an additional 10 m on either end of the rectangle to represent the 10 m distance observers were allowed to wander off the point count station for flock observation. The width of this rectangular area was 20 m, because observers were allowed to wander off 10 m on each side of the travel route between point count stations to observe flocks. L is thus expressed as:

$$L = (n-1) \times 250 + 10 + 10 \text{ m}$$

The overall land area surveyed for flocks within each land-cover type (represented as A_f) is thus expressed as:

$$A_f = L \times 20 \text{ m}^2$$

$$= 20 \times [(n-1) \times 250 + 20] \text{ m}^2$$

With known A_{pc} and A_f, we were thus able to mathematically express the expected number of individuals/groups detected during point count and flock observation using parameters pertaining to population density and detection probability, and estimate these parameters by fitting the expressions to observed data using the maximum likelihood approach.

For the analysis of household interview data, we first calculated the annual per ha sale and cost (in US$ and labor days) for each household, GFGP forest type and tree species, based on forest product yield and unit price, length of production cycle, and various aspects of production cost during initial forest establishment and subsequent maintenance. We in turn calculated the annual per ha profit by subtracting all available aspects of production cost from

the annualized gross rents, including initial cost of forest establishment, annual maintenance cost as directly reported by households, and harvest cost. We applied a discount rate of 5% (r=0.05) to production sale, cost and labor input based on the 2015 one-year lending rate of the People's Bank of China (range 4.35-6% [27]). For data from households with mixed forests that provided information on more than one species, such calculation involved all species for which data were available through weighted sum based on the production area of each species. We did not use mixed forest-owning households that provided production or labor estimates for only one tree species to estimate the profit or labor intensity of mixed forest.

Among the three main tree species used in GFGP in the study region, bamboo is generally harvested every year, while eucalyptus and Japanese cedar have a harvest (i.e. clear-cut) cycle of around seven years and 20 years, respectively. For households that did not report the number of years it took for bamboo to start producing, we assumed harvest started in the third year after forest establishment and used a 20 year time span of which production happened in 18 years to calculate average annual sale. For bamboo and eucalyptus forests, we calculated the annualized net rent and labor input over the harvest cycles (harvest cycle of eucalyptus was directly reported by respondent households) with the 5% discount rate. For Japanese cedar forest, forest production typically entailed a one-time clear-cut at the end of each production cycle, with 2-3 rounds of selective harvest before the final clear-cut. We assumed that selective harvesting of these forests happened at the mid-point of the reported production cycle. The net rent was therefore a summation of the net present value (NPV) for the clear-cut (full production cycle) and the NPV of the reported selective harvest. As with bamboo and eucalyptus, we applied the discount rate of 5% to calculate annualized net rent and labor input.

We conducted all analyses using multiple linear models. For the self-reported percentage of household income contributed by forest production, visualization of the distribution of the self-reported percentages suggested possible outliers toward the high end of the values. We thus used the Tietjen-Moore test for outliers [19] to identify estimates with excessively high percentages ($P < 0.05$) and eliminated these outliers. This resulted in the removal of four data points (all 100% despite very small household land-holding size). We then tested for the difference in this percentage among different types of GFGP forest, but additionally included forest area as a nuisance covariate because of its obvious relationship to the response variable. For annual per ha profit and labor intensity, we included forest type as the only covariate. To avoid the specific discount rates driving our conclusions, we additionally used alternative discount rates (r=0, 0.0125, and 0.025; one-year interest rate for personal saving was 1.35-1.75% as of October 24th, 2015 [27]) for the full set of analysis. Results based on all discount rates are presented in Supplementary Table 6.

Supplementary References

1. Chao, A. & Jost, L. Coverage-based rarefaction and extrapolation: Standardizing samples by completeness rather than size. *Ecology* **93,** 2533–2547 (2012).
2. Liu, J., Li, S., Ouyang, Z., Tam, C. & Chen, X. Ecological and socioeconomic effects of China ' s policies for ecosystem services. *Proc. Natl. Acad. Sci.* **105,** 9477–9482 (2008).
3. Yin, R., Yin, G. & Li, L. Assessing China's ecological restoration programs: what's been done and what remains to be done? *Environ. Manage.* **45,** 442–453 (2010).
4. Song, X., Peng, C., Zhou, G., Jiang, H. & Wang, W. Chinese Grain for Green Program led to highly increased soil organic carbon levels: A meta-analysis. *Sci. Rep.* **4,** 1–7 (2014).
5. Delang, C. O. & Yuan, Z. *China' s Grain for Green Program - a review of the largest ecological restoration and rural development program in the world*. Springer Press (2015).
6. Yin, R. & Zhao, M. Ecological restoration programs and payment for ecosystem services as integrated biphysical and socioeconomic processes – China's experience as an example. *Ecological Economics* **73,** 56-65 (2012).
7. Trac, C. J., Schmidt, A. H., Harrell, S. & Hinckley, T. M. Is the Returning Farmland to Forest Program a success? Three case studies from Sichuan. *Environmental Practice* **15,** 350-366 (2013).
8. Alldredge, M. W., Pollock, K. H., Simons, T. R., Collazo, J. A. & Shriner, S. A. Time-of-detection method for estimating abundance from point-count surveys. *Auk* **124,** 653–664 (2007).

9. Bartholomew, C. S. & Prowell, D. Pan compared to Malaise trapping for bees (Hymenoptera: Apoidea) in a longleaf pine savanna. *J. Kansas Entomol. Soc.* **78,** 390–392 (2005).
10. Ivanova, N. V., Dewaard, J. R. & Hebert, P. D. N. An inexpensive, automation-friendly protocol for recovering high-quality DNA. *Mol. Ecol. Notes* **6,** 998–1002 (2006).
11. Françoso, E. & Arias, M. C. *Cytochrome c oxidase I* primers for corbiculate bees: DNA barcode and mini-barcode. *Mol. Ecol. Resour.* **13,** 844–850 (2013).
12. Pons, J. *et al.* Sequence-Based Species Delimitation for the DNA Taxonomy of Undescribed Insects. *Syst. Biol.* **55,** 595–609 (2006).
13. Bouckaert, R., Heled, J., Kühnert, D., Vaughan, T., Wu, C. H., Xie, D., Suchard, M. A., Rambaut, A., Drummond, A. J. BEAST 2: a softward platform for Bayesian evolutionary analysis. *PLoS Computational Biology* **10,** e1003537 (2014).
14. Guindon, S. & Gascuel, O. A. simple, fast, and accurate algorithm to estimate large phylogenies by maximum likelihood. *Systematic Biology* **52,** 696-704 (2003).
15. Darriba, D., Taboada, G. L., Doallo, R. & Posada, D. jModelTest 2: more models, new heuristics and parallel computing. *Nature Methods* **9,** 772 (2012).
16. Rambaut, A. & Drummond, A. J. Tracer version 1.6.0. (2007).
17. Ezard, T., Fujisawa, T. & Barraclough, T. splits: SPecies' LImits by Threshold Statistics. R package version 1.0-19/r51. http://R-Forge.R-project.org/projects/splits/ (2014).
18. R Core Team. R: A language and environment for statistical computing. R Foundation for Statistical Computing, Vienna, Austria. URL https://www.R-project.org/ (2015).
19. Tietjen, G. L. & Moore, R. H. Some Grubbs-Type Statistics for the Detection of Outliers, *Technometrics* **14,** 583-597 (1972).

20. Chao, A. *et al.* Rarefaction and extrapolation with Hill numbers: A framework for sampling and estimation in species diversity studies. *Ecol. Monogr.* **84,** 45–67 (2014).

21. Anderson, M. J. A new method for non-parametric multivariate analysis of variance. *Austral Ecology* **26,** 32-46 (2001).

22. Anderson, M. J. & Walsh, D. C. PERMANOVA, ANOSIM, and the Mantel test in the face of heterogeneous dispersions: What null hypothesis are you testing? *Ecological Monographs* **83,** 557-574 (2013).

23. del Hoyo, J., Elliott, A. & Christie, D. Handbook of the Birds of the World. Lynx Edicions (1992-2013).

24. MacKinnon, J. R., Phillipps, K. & He, F. Q. A field guide to the birds of China. Oxford University Press (2000).

25. Burnham, K. P. & Anderson, D. R. Model selection and multimodel inference: a practical information-theoretic approach. Springer Science and Business Media (2002).

26. Royle, J. A. N-mixture models for estimating population size from spatially replicated counts. *Biometrics* **60,** 108–115 (2004).

27. Index Mundi. Lending interest rate of China. Available online: http://www.indexmundi.com/facts/china/lending-interest-rate (accessed on November 7th, 2015). (2015).

28. Peng, H., Cheng, G., Xu, Z., Yin, Y., Xu, W. Socio, economic, and ecological impacts of the “Grain for Green” project in China: a preliminary case in Zhangye, Northwest China. *Journal of Environmental Management* **85,** 774-784 (2007).

29. Wang, G., Yang, Z., Zha, T., Zhang, Z., Lv, Z., Zhang, X., Wang, H. & Zhu, J. Water-holding capacity of different forestlands 20 years after converted from farmland in Loess region, western Shanxi Province. *Journal of Beijing Forestry University* **37,** 88-95 (2015).
30. Wang, T. Measurement of ecotone width between agro- forest ecosystems affected by Grain for Green Program. *Yunnan: Yunnan University* 1-65 (2012).
31. Wang, L. Ecological health assessment of artificial forests by grain for green in Minshan. *Beijing: Beijing Forestry University* 1-155 (2004).
32. Yang, X. 甘肃天祝退耕还林不同配置模式生态功能的综合评价. *Gansu Science and Technology* **22,** 13-16 (2006).
33. Ling, A. Monitoring analysis and value assessment to ecological benefit of Conversion of Farmland to Forest in Wushan County of Gansu. *Journal of Gansu Forestry Science and Technology* **37,** 47-50 (2012).
34. Zhen, J. & Chen, Y. Vegetation indices and the applications in monitoring tillage reverting to woodland or grassland ecosystems. *Remote Sensing Technology and Application* **21,** 41-48 (2006).
35. Kong, X. & Wang, K. Eco-economic effect of Returning Cultivated Land to Forestry Project in karst area - taking Hechi City as an example. *Acta Agriculturae Jiangxi* **21,** 138-142 (2009).
36. Li, B., Huang, C., Zhuang, J., Lai, J. & Chen, C. Research on the runoff characteristics of small watershed under the reafforestation patterns in Northwest Guangxi. *Journal of Anhui Agri. Sci.* **37,** 3082-3084 (2009).
37. Wei, Q. 象州县 2002 年度退耕还林工程建设及效益分析. *Central South Forest Investigation and Planning* **22,** 28-30 (2003).

38. Zhang, X. & Cui, Y. Eco-division and adaptive models for returning farmland to forest project in Guizhou karst upland. *Guizhou Forestry Science and Technology* **33,** 46-52 (2005).

39. Yuan, H. A Study on evaluation of the social, economic, ecological benefits of the consolidation phase of the Program of Converting Farmland to Forestry Land in Dejiang County. *Guizhou: Guizhou University* 1-71 (2009).

40. Wang, Y. The quantitative evaluation research on eco- efficiency for conversion of cropland to forest in Liping County Guizhou. *Beijing: Beijing Forestry University* 1-85 (2012).

41. Zhou, S., Yin, Y., Xu, W., Ji, Z., Caldwell, I. & Ren, J. The costs and benefits of reforestation in Liping County, Guizhou Province, China. *Journal of Environmental Management* **85,** 722-735 (2007).

42. Wang, S. 大河镇退耕还林柳杉工程造林效果初步研究. *Guizhou: Guizhou University* 1-68 (2008).

43. Yang, A., Wang, Y. & Gan, Q. Analysis of main afforestation models for Returning Farmland to Forest in Majiang County. *Journal of Anhui Agricultural Sciences* **36,** 3335-3336 (2008).

44. Zhang, X., Yu, S., Long, X., Zhou, H. & Yang, C. Effects of water and soil conservation by intensive managements in forest converted from farm land. *林业科技开发* **22,** 30-32 (2008).

45. Mei, Z. & Xiong, K. A study on the dynamic characteristics of soil erosion and eco-benefit evaluation in karst region – a case study on Qingzhen demonstrating site of Returning Farmland to Wood (Grass), Guizhou. *Carsologica Sinica* **2,** 136-143 (2003).

46. Zhang, X., Lian, B., Yin, J., Liu, Y. & Cui, Y. Study on influence of different land use type on soil qualities in Plateau Karst depression. Journal of Anhui Agricultural Science **38,** 5771-5775 (2010).

47. Long, J., Deng, Q., Jiang, X. & Liu, F. Effects of different de-farming and reafforestation patterns on changes of soil fertility quality in karst region of southwestern China. *Chinese Journal of Applied Ecology* **16,** 1279-1284 (2005).

48. Yao, P. & Wen, Y. Comprehensive benefits evaluation on the project of conversion of cropland to forest in Chengde, Hebei Province. *Journal of Arid Land Resources and Environment* **27,** 47-53 (2013).

49. Cheng, Z., Yang, F., Zhang, Y. & Wu, G. Evaluation of ecological benefit of Returning Farmland to Forestry Projects in Nanzhao County of Henan Province. *Meteorological and Environmental Sciences* **37,** 64-69 (2014).

50. Wu, D., Dai, Y., Li, S., Han, M. & Wu, Y. Evaluation on benefits of different managements for the Grain for Green Project in southwest Hubei. *Forestry Science, Technology and Development* **25,** 112-115 (2011).

51. Zhao, X., Tian, K. & Chen, D. Study on afforestation model of converting land to forest. *Hubei Forestry Science and Technology* **142,** 5-9 (2006).

52. Chen, L., Xiao, W., Tang, W., Pan, L. & Shi, Y. Study on soil organic carbon under several reafforestation patterns in the Three Gorges Reservoir Area. *Scientia Silvae Sinicae* **43,** 111-114 (2007).

53. Yang, X. The benefits evaluation and impact research on the land conversion program in Chinese Western Region. *Beijing: Beijing Forestry University* 1-181 (2004).

54. Zeng, L., Huang, Z., Xiao, W. & Tian, Y. Nitrogen and phosphorus loss in different land use types and its response to environmental factors in the Three Gorges Reservoir Area. *Environmental Science* **33,** 3390-3396 (2012).

55. Tian, Y., Li, X., Liu, Z., Li, S., Yao, M. & Xia, H. The strengthen effects on the anti-rush ability of the soil of the returning land for farming to forestry. *Hunan Forestry Science and Technology* **33,** 28-32 (2006).

56. Zhou, G., Tian, Y., Chen, G., Fu, Z. & Bao, W. Runoff and sediment yield on slope land in initial stages of conversion of cropland to forest in Northwestern Hunan Province. *Science of Soil and Water Conservation* **7,** 118-122 (2009).

57. Zhou, W. The research on ecological benefit for conversion of cropland to forest in Cili County Hunan Province. *Beijing: Beijing Forestry University* 1-74 (2011).

58. Tian, D., Yin, G., Fang, X., Xiang, W. & Yan, W. Carbon density, storage and spatial distribution under different "Grain for Green" patterns in Huitong, Hunan Province. *Acta Ecologica Sinica* **22,** 6297-6308 (2010).

59. Yin, G. Eco-environmental effects under different 'Grain for Green' patterns in Huitong, Hunan Province. *Hunan: Central South University* 1-144 (2010).

60. Yin, G., Tian, D., Fang, X., Xiang, W. & Deng, X. Studies on biomass of different young forests converted from farm land in Huitong, Hunan Province. *Journal of Central South University of Forestry& Technology* **30,** 9-14 (2010).

61. Zhang, G., Tian, D., Fang. X. & Xiang, W. Distribution characteristics of soil organic carbon in Huitong as affected by different afforestation models for Conversion of Cropland to Forestland. *Journal of Central South University of Forestry &Technology* **28,** 8-12 (2008).

62. Li, S., Li, X., Tian, Y., Tao, J., Zhang, Y., Yao, M. & Dong, C. The investigation and analysis on the achievements and effects of the ecological type and economical ecological type forestry mode of the Returning Land for Farming to Forestry Project in Hunan. *Hunan Forest Science and Technology* **33,** 17-20 (2006).
63. Li, M., Huang, Z., Yang, X., Chen, H. & Yang, H. The afforestation mode of ornamental plants of returning land for farming to forestry in Nanyue District. *Hunan Forest Science and Technology* **33,** 70-73 (2006).
64. Deng, C. Assessment on Benefits of the Project of Conversion Cropland to Forest in the Ecosystems core area of Wuling Mountains. *Hunan: Central South University of Forestry and Technology* 1-53 (2013).
65. Zhao, X., Hao, F., Liu, L., He, S., Deng, M. & Zhao, W. Afforestation models of Returning Crop Plots to Forestry in Ordos Area. *Journal of Desert Research* **22,** 499-505 (2002).
66. Liu, W., Wang, F., Ke, G., Wang, Y., Guo, S. & Fan, C. Effects of converting cultivated land into forest land on the characteristics of soil organic carbon in limestone mountain area in Ruichang, Jiangxi. *Chinese Journal of Applied Ecology* **22,** 885-890 (2011).
67. Li, T., Liu, W., Guo, S., Ke, G., Zhang, Z., Xiao, X. & Liu, W. Community traits of soil fauna in forestlands converted from cultivated lands in limestone red soil region of Ruichang, Jiangxi Province of China. *Chinese Journal of Applied Ecology* **23,** 910-916 (2012).
68. Liu, Y., Song, N. & Wang, L. Case study on pattern of Converting Cropland to Forest and Grassland in Loess Plateau - taking Yuanzhou District in Ningxia as an example. *Journal of Desert Research* **27,** 419-424 (2007).

69. Chang, G., Zhao, W., He, K. & Shi, C. Water consumption of forests in project area of returning farmland to forests in Datong county, Qinhai province. *Science of Soil and Water Conservation* **3,** 58-65 (2005).

70. Gao, G., Li, W., Zhou, X., Jia, J., Shi, C. & Li, S. Synthetic assessment on ecology service function of conversion cropland to forest in Datong county of Qinghai province. *Journal of Northwest A and F University (Nat. Sci. Ed.)* **35,** 130-134 (2007).

71. Gao, C., Wang, D., Wang, S., Luo, X. & Zhao, Y. Development situation and countermeasure of returning farmland to forestry follow-up industries in Daxian. *Journal of Hebei Agricultural Sciences* **12,** 90-92 (2008).

72. He, K., Li, S., Shi, C. & Zhou, X. Tree productivity and water production potential in Returning Farmland to Forest Project in Datong County, Qinghai Province. *Journal of Beijing Forestry University* **27,** 28-32 (2005).

73. Jia, J., Li, W., Gao, G., Li, D., Li, Y. & Zhou, X. Species diversity of different species arrangement models in Converting Cropland to Forest in Datong, Qinghai. *Journal of Northwest Forestry University* **21,** 1-6 (2006).

74. Li, W. Study on ecological of different species arrangement models in converting cropland to forest in Datong of Qinghai. *Beijing: Beijing Forestry University* 1-117 (2005).

75. Li, S. Studies on ecological effect of converting cropland to forest in Datong high-cold zone of Qinghai. *Beijing: Beijing Forestry University* 1-167 (2006).

76. Li, W., He, Y., Zhang, W. & Zhou, X. Evaluation of influence of Changing Slope Farmland into Forest and Grassland on soil quality in Beichuanhe Watershed. *Research of Soil and Water Conservation* **12,** 1-3 (2005).

77. Lv, F. The characteristics of habitat succession and its evaluation of different vegetation in Datong county. *Beijing: Beijing Forestry University* 1-135 (2007).

78. Lv, F., Han, Z., Zhang, W. & Zhou, X. Study on anti-scourability of Converting Cropland into Forest soil in Datong County, Qinghai Province. *Acta Agriculturae Boreali - Sinica* **20,** 85-90 (2005).

79. Qiao, W., Huo, G., Zhang, W. & Wu, Y. 青海大通脑山区退耕还林还草地土壤质量演变的初步研究. *青海环境* **18,** 49-52 (2008).

80. Shi, C. Study on the ecological benefits in high- cold areas on Loess Plateau. *Beijing: Beijing Forestry University* 1-159 (2007).

81. Zheng, J., Gao, G., Lu, F., Shi, C., Kang, Y. & Lei, X. Soil quality evolution on converted farmland in high-mountain of Datong County in Qinghai Province. *Bulletin of Soil and Water Conservation* **27,** 6-10 (2007).

82. Dong, X. Ecological effects of different models of conversion of farmland to forest in hilly Loess Plateau in Qinghai Province. *Forest Resources Management* **4,** 71-75 (2011).

83. Bao, F., Yan, J. & Sun, H. Effect evaluation of reforesting application on farmland in the alpine farming-pastoral ecotone. *Research of Soil and Water Conservation* **15,** 151-155 (2008).

84. Dong, X. Effects of Grain-for-Green on soil quality in Huangshui River Basin of Qinghai Province. *Bulletin of Soil and Water Conversion* **31,** 45-48 (2011).

85. Yin, H. The study of implementation and development strategy of the conversion of cropland to forest in Shaanxi Province. *Shaanxi: Northwest A and F University* 1-49 (2010).

86. Liu, Z., Du, Y., Xu, L., Yang, G. & Feng, Y. Analysis on economical effect of project of "returning farmland to forest and grassland" in the Loess Hilly and Gully Area: a case study in Ansai County. *Acta Ecologica Sinica* **28,** 1476- 1482 (2008).
87. Peng, W., Zhang, K., Chen, Y. & Yang, Q. Research on soil quality change after returning farmland to forest on the Loess sloping croplands. *Journal of Natural Resources* **20,** 272-278 (2005).
88. Cheng, R. & Zhang, L. Investigation and research on returning cropland to forestry in western China - taking Dongjia Village, Shitan Town, Liquan County as an example. *Inner Mongolia Forestry Investigation and Design* **28,** 3-7 (2005).
89. Liang, W., Bai, C., Sun, B., Hao, D. & Qi, J. Study on soil moisture physical properties of returning land for farming to forestry (grass) region in gullied rolling Loess area. *Soil and Water Conservation in China* **3,** 17-18 (2006).
90. Wang, Y. Effects of different Grain for Green mode on the soil and growth state of plant communities in Pianguan District. *Shanxi: Shanxi Agricultural University* 1-47 (2014).
91. Zhang, G. The research about the Project of Farmlands to Forests ecological benefit in Pinglu County. *Shanxi: Shanxi Agricultural University* 1-54 (2014).
92. Zhang, P. Study on vegetation restoration after Conversion of Farmland to Forests in Hutou Mountain in Dazhai. *Shanxi: Shanxi University* 1-47 (2007).
93. Zhang, X. Research on establishing benefit evaluation index system of massive sloping land conversion program in China sampled in key district of Zhongyang County, Shanxi. *Beijing: Beijing Forestry University* 1-107 (2009).

94. Hao, Y., Wang, J. & Li, L. Analysis of the Economic Profit of Different Models in Beichuan, under Sloping Land Conversion Program. *Journal of Sichuan Agricultural University* **23,** 313-317 (2005).

95. Li, X. Study on theoretical foundation and technology in Converting Farmland to Forest. *Sichuan: Sichuan Agricultural University* 1-180 (2004).

96. Wan, X. Vegetation restoration and its effects on soil after converting agricultural lands to forest. *Sichuan: Sichuan Agricultural University* 1-59 (2003).

97. Yang, Z. & Xie, Y. Research on ecological problem and technological model of Returning Land for Farming to Forestry in Jinshajiang Dry Heat Valley. *Sichuan Forestry Exploration and Design* **4,** 28-33 (2003).

98. Ji, X., Wang, Y., Yu, X., Zheng, W., Li, W. & Tang, L. 德阳市西部山区退耕还林后林下经济发展对策. *Journal of Sichuan Forestry Science and Technology* **33,** 112-115 (2012).

99. Li, D. The research of soil and fine root respiration of forest mode in converting farmlands to forest. *Sichuan: Sichuan Agricultural University* 1-65 (2007).

100. Xu, Y. Study on soil physical and chemical properties and soil enzyme dynamics of four de-farming and reafforestation on patterns. *Sichuan: Sichuan Agricultural University* 1-53 (2007).

101. Wang, T. Research on comprehensive benefit evaluation of the grain for green project in Hongya County. *Beijing: Beijing Forestry University* 1-153 (2013).

102. Yan, D. Study on soil physical and chemical properties and soil enzyme dynamics of four de-farming and reafforestation patterns in high precipitation area of western China. *Sichuan: Sichuan Agricultural University* 1-55 (2008).

103. Zhang, H. 中国西部地区退耕还林政策绩效评价与制度创新. *Beijing: Minzu University of China* 1-158 (2009).

104. Zhou, X. The research on ecological benefit evaluation of conversion from cropland to forest in Hongya County Sichuan Province. *Beijing: Beijing Forestry University* 1-93 (2012).

105. Li, J., Liu, Y. & Sun, L. Positive effects of education migration to the Land Conversion Program in Liangshan, Sichuan Province. *Science Technology and Engineering* 3: 494-499 (2003).

106. Li, Y., Liu, Y., Long, H. & Wang, J. Local responses to macro development policies and their effects on rural system in China's mountainous regions: the case of Shuanghe Village in Sichuan Province. *Journal of Mountain Science* **10,** 588-608 (2013).

107. Wang, Y. The evaluation on the effect of water and soil conservation for different vegetation restoration models in the area of converting agricultural land into forest and pasture. *Sichuan: Sichuan Agricultural University* 1-50 (2002).

108. Mou, J., Pan, P., Zhou, S., Zhao, H. & Tang, C. Operation patterns of vegetation restoration in Returning the Grain Plots to Forestry in Sichuan Province. *Journal of West China Forestry Science* **34,** 63-67 (2005).

109. Li, C. Efficiency assessment of Conversion Cropland to Forest and Grassland Project in the inland river of Hungriness Valley. *新疆：新疆农业大学* 1-50 (2004).

110. Li, C., Dai, L. & Wu, X. Assessment of eco-economic efficiency of converting cropland to forest and grassland in the middle-lower reaches of Tarim River. *Hubei Agricultural Sciences* **51,** 4440-4442 (2012).

111. Zhao, P., Kong, W. & He, L. Land conservation afforestation modes and technology in arid-hot valley of Jinsha River. *Shaanxi Forest Science and Technology* **5,** 29-34 (2014).

112. Zhou, L. 泸水县退耕地种植模式探讨. *Biotech world* **5,** 28 (2015).

113. Su, C., Shang, Y., Hong, Y. & Li, J. Improving soil effects after different returning farmland to forest in Pu'er City of Yunan. *SWCC* **11,** 46-50 (2013).

114. Su, C. & Shang, Y. Study on hydrological effect of different modes of returning farmland to forest in mountainous area of Lancang River Basin. *Yangtze River* **45,** 36-40 (2014).

115. Zhong, X. Evaluation of ecological effect of conversion farmland to forest in Qiaojia County of Yunnan Province. *Beijing: Beijing Forestry University* 1-59 (2012).

116. Chen, J. 滇西北金沙江峡谷退耕林地昆虫多样性快速评估 (Doctoral Dissertation). *Southwest Forestry University, Kunming, Yunnan* (2008).

117. Li, P., Chen, Q., Chang, E., Yin, A. & Bi, B. Preliminary study on land capacity change of different Returning Cultivated Land to Forest model soils at karst mountains desertification area in southeastern Yunnan. *Research of Soil and Water Conservation* **15,** 65-68 (2008).

118. Zeng, Y. 镇雄县退耕还林的效益分析. *Forest Inventory and Planning* **31,** 197-200 (2006).

119. Yu, P. Stage achievement investigation and benefit evaluation of Return Cultivated Land to Forest - to Nangang Town, Shucheng County as an example. *Anhui Agricultural Science Bulletin* **17,** 218-221 (2011).

120. Zhang, B., Zhuang, J., Zhang, J. & Wang, R. Evaluation of the function of reforestation on soil loss control in the Dabie Mountains, China. *Bioinformatics and Biomedical Engineering (iCBBE),* 4th International Conference (2010).

121. Kang, M. The ecological benefit monitoring and evaluation of returning farmland to forest project in Hechuan district Chongqing city. *Beijing: Beijing Forestry University* 1-72 (2014).

122. Yao, P., Chen, X., Zhou, Y., Zhao, W., Lu, M. & Tu, J. Carbon sequestration potential of the major stands under Grain for Green Program in southwest China in the next 50 years. *Acta Ecologica Sinica* **34,** 3025-3037 (2014).

123. Xin, Z., Yu, X., Zhang, M., Li, Q. & Li, H. Soil nutrient characteristics under different land use types in a gully-hilly region of the Loess Plateau. *Arid Zone Research* **29,** 379-384 (2012).

124. Zhao, Y., Yang, Y., Sun, B., Guo, J. & Zhou, W. Research of the effect on the physical properties of different' Conversion of Farmland to Forest' practices -take Dingxi City of Gansu Province as an example. *Chinese Agricultural Science Bulletin* **25,** 99-105 (2009).

125. Li, Y., Zhang, J., Tian, N. & Zhao, R. Effects on soil microelements of Grain for Green Project in semi-arid area of Loess Plateau. *Bulletin of Agricultural Mechanics 农业机械学报* **8,** 017 (2015).

126. Zhang, L., Xie, Z., Zhao, R. & Wang, Y. The impact of land use change on soil organic carbon and labile organic carbon stocks in the Longzhong region of Loess Plateau. *Journal of Arid Land* **4,** 241-250 (2012).

127. Zhao, S., Su, J., Wu, J., Yang, Y. & Liu, N. Changes of soil aggregate organic carbon during process of vegetation restoration in Ziwuling. *Journal of Soil and Water Conservation* **20,** 114-117 (2006).

128. Chen, B., Chang, Z., Qiao, J., Chen, F. & Fan, B. Estimation of relative ecological value of returning farmland to forest &mountain enclosure: a case of Yongxing Township of Jingyuan County, Gansu Province. *Arid Land Geography* **36,** 1144-1152 (2013).

129. Ma, Y. & Fan, S. Eco-economic effect of actualizing de-farming and reafforestation policy in desertification areas: taking Minqin County as a case. *Journal of Natural Resources* **20,** 590-596 (2005).

130. Chen, Y. Study on quantitative monitoring and evaluation for converting farmland to forest program. *Beijing: Beijing Forestry University* 1-153 (2006).

131. Shi, P., Feng, X., Song, X. & Yu, J. Evaluation on the effects of implementing the policy of Returning Land from Farming to Forestry and Grassplot on farmers' pure economic returns – a case study in four test regions of Gansu Province. *Arid Zone Research* **23,** 459-465 (2006).

132. Shi, W., Wu, T. & Zhang, L. 对天水市退耕还林（草）实施情况的调查与思考. *Forestry Resource Management 林业资源管理* **1,** 22-24 (2002).

133. Ma, H., Yong, Y. & Liu, Z. Comprehensive evaluation of returning cropland to forest and grassland in the arid and semi- arid region. *Pratacultural Secience* **29,** 1359-1367 (2012).

134. Zhen, J. & Chen, Y. Vegetation indices and the applications in monitoring tillage reverting to woodland or grassland ecosystems. *Remote Sensing Technology & Application* **21,** 41-48 (2006).

135. Wei, X. Investigation and study on vegetation- effect in different vegetation restoration patterns of returning land for farming to forestry in Guangxi. *Guangxi: Guangxi University* 1-90 (2008).

136. Hu, D., Zhuang, J., Wang, W. & Liang, Y. Diversity and biomass in the Costanea mollissima forest under conversion cultivated land into forests model. *Journal of Anhui Agricultural Sciences* **42,** 6749-6751 (2014).

137. Niu, H. Study on the comprehensive benefits evaluation of different economic forest by returning farmland to forestry in northwest Guangxi. *Guangxi: Guangxi University* 1-71 (2013).

138. Xie, W., Xie, S., Jiang, G., Lai, J. & Wei, X. Vegetational diversity and biomass in *Juglans regia* forest under Conversion Cultivated Land into Forests Model. *Guizhou Agricultural Sciences* **38,** 177-179 (2010).

139. Lu, S., Peng, W., Song, T., Zeng, F., Du, F. & Wang, K. Soil microbial properties under different grain-for-green patterns in depressions between karst hills. *Acta Ecologica Sinica* **32,** 2390-2399 (2012).

140. Ouyang, Z. Study on ecological effects of different models of converting cropland to forest and grass in Karst peak- cluster- depression region. *Hunan: Hunan Agricultural University* 1-118 (2010).

141. Song, T., Peng, W., Zeng, F., Wang, K., Liu, L., Lu, S. & Du, H. Soil ecological effects of converting cropland to forest and grassland in depressions between Karst hills. *Acta Pedologica Sinica* **48,** 1219-1226 (2011).

142. Yuan, H., Su, Y., Zheng, H., Huang, D. & Wu, J. Distribution characteristics of soil organic carbon and nitrogen in peak cluster depression landuse of karst region. *Chinese Journal of Ecology* **26,** 1579-1584 (2007).

143. Zeng, F. & Wang, K. Effects of' grain-for-green' models in karst regions in Northwest Guangxi. *Rural Eco-Environment* **21,** 18-22 (2005).

144. Yu, M. & Sun, B. Environmental effect of returning farmland to forest and its ecological value estimation in Pingguo County, Guangxi Zhuang Autonomous Region. *Acta Agriculturae Zhejiangensis* **24,** 114-119 (2012).

145. Zhao, R., Sun, B., Yu, M. & Guo, C. Characteristics of carbon storage in forests of Grain for Green Project in Pingguo County, Guangxi Zhuang Autonomous Region. *Bulletin of Soil and Water Conservation* **35,** 350-353 (2015).

146. Zuo, Z. Ecological benefits of bamboo plantation in the conversion program – a case study of *Demdroclamus farinosus* in Chishui region. Master's thesis, Chinese Academy of Forestry (2007).

147. Yuan, T. Achievements, effects and sustainable development countermeasures on the project of Returning Farmland to Forestland in Dejiang County. *Journal of Anhui Agricultural Science* **35,** 11599-11600 (2007).

148. He, Y. & Liu, J. Study on the vegetation recovery benefit from different conversions of farmland to forests models in Huajiang Area of low-heat river valley. *Journal of Anhui Agri. Sci.* **36,** 1439-1440 (2008).

149. Zhang, X. & Ren, C. Monitoring and evaluation on the ecological and economic benefit of Returning Farmland to Forest demonstration area in karst gorge in Guizhou. *Guizhou Forestry Science and Technology* **34,** 42-46 (2006).

150. Jiao, S. & Ai, Q. Study on the carbon sink effects in Conversion of Farmland to Forest Project in karst drainage basin. *Journal of Anhui Agricultural Sciences* **39,** 17402-17404 (2011).

151. Wang, J., Fan, J., Wang, L. & Shi, Z. Soil respiration and assessment of the carbon budget in the process of Returning Farmland to Forest and Grassland under different land use patterns. *Journal of Agro-Environment Science* **30,** 2024-2031 (2011).

152. Wang, Y., Sun, B., Zhao, Y., Zhou, X. & Zhong, X. Comparison of vegetative biomass in different configuration modes for reforestation of cultivated land in Liping County of Guizhou Province. *Hunan Agricultural Sciences* **11,** 120-122 (2011).

153. Gou, X., Wan, X., Liao, X. & Liu, X. Difference of organic carbon content in soil of three typical plantations in Southwest China. *Guizhou Agricultural Sciences* **43,** 151-155 (2015).

154. He, X., Chen, X., Zhang, R., Li, X., Wang, Y. & Jiang, L. Variation of soil organic carbon storage in fir forests under the Grain for Green Program - take Sanjiangkou, Xingyi of Guizhou Province as an example. *Forest Resources Management* **2,** 95-99 (2015).

155. Liu, F., Huang, C., He, T., Qian, X., Liu, Y. & Luo, H. Roles of reducing phosphorus loss of surface runoff from yellow soil in hilly areas by defarming and reafforestation. *Journal of Soil and Water Conservation* **16,** 20-23 (2002).

156. Yao, Q., Lu, G., Du, J. & Tan, X. Evaluation of ecological benefits from returning land for farming to forestry in Chengde City. *Journal of Agricultural University of Hebei* **32,** 57-61 (2009).

157. Li, D., Zhang, Y., Xu, Z. & Zhang, X. Investigation on the stand growth status of returning farmland to forest and its policy suggestion. *Journal of Anhui Agricultural Sciences* **36,** 8595-8598 (2008).

158. Cui, L. & Li, Z. Effect of Returning Farmland to Forest (Grass) on phosphorus runoff of slope land. *Research of Soil and Water Conservation* **14,** 272-274 (2007).

159. Jiao, Y., Zhang, Y., Meng, D., Zhang, S. & Lu, G. Analysis of benefits on returning arable land to forest and grassland in Qianxi. *Hebei Journal of Forestry and Orchard Research* **22,** 271-273 (2007).

160. Liu, J. 伐根佳节造林技术在邢台县退耕还林工程中的应用. *Modern Agricultural Science and Technology* **2,** 35 (2015).

161. Yang, M., Yang, X., Ma, Y., Han, M., Jiang, F., Xu, J., Xu, Z. & Chen, X. Dynamics of topical erodibility during converting farmland to forest in black soil. *Journal of Northeast Forestry University* **42,** 98-101 (2014).

162. Tian, Y., Qu, Y., Man, X., Liu, B., Zhang, W. & Yu, S. Effect of soil and water conservation measures on physical and chemical properties of black soil erosion area. *Journal of Northeast Forestry University* **39,** 84-88 (2011).

163. Liu, J., Li, S., Ouyang, Z., Tam, C. & Chen, X. Ecological and socioeconomic effects of China's policies for ecosystem services. Proceedings of National Academy of Sciences **105,** 9477-9482 (2008).

164. Wang, X. The development countermeasures of conversion of farmland to forests in Lushan County. *Henan: Henan Agricultural University* 1-51 (2008).

165. Han, L. The Returning Farmland to Forests' main ecology effect and the value research in the hilly area of west part in Henan Province - take Luoning County as an example. *河南：河南农业大学* 1-42 (2008).

166. Han, X. Research on ecological effect and mechanism of forestry (grass) project in Loess plateau. *Shaanxi: Northwest A and F University* 1-174 (2008).

167. Han, L., Zheng, J., He, K., Zheng, G. & Wang, Z. Effect of Returning Farmland into Forest on soil physicochemical properties in Datong County of Qinghai Province. *Research of Soil and Water Conservation* **16,** 100-104 (2009).

168. Leng, B., Han, L., Zhang, M. & Li, X. Value and effect of carbon sequestration and oxygen release on returned cropland to forest in Luoning County. *Journal of Henan Forestry Science and Technology* **29,** 6-8 (2009).

169. Yu, H. 信阳市浉河区退耕还林工程后续产业发展成效与主要做法. *Modern Agricultural Science and Technology* **15,** 215-217 (2014).

170. Zhang, M. The development countermeasures of conversion of farmland to forests in Xin'an County. *Henan: Henan Agricultural University* 1-56 (2005).

171. Tu, W. & Zhang, M. Humble opinion on results consolidation of Grain for Green Project in Hubei Province. *Hubei Forestry Science and Technology* **169,** 162-164 (2011).

172. Liao, J., Song, Z., Lv, Z., Xiang, C. & Wang, Q. 赤壁市退耕还林工程效益分析. *Hubei Forestry Science and Technology* **42,** 68-71 (2013).

173. Ding, L., Huang, C., Zhang, S. & Zheng, S. Analysis of the situation of returning the grain plots to forestry (grass) in Enshi City. *Journal of Hubei Institute for Nationalities (Natural Science Edition)* **21,** 36-40 (2003).

174. Liu, H., Peng, F., Ding, J. & Li, S. 黄陂区退耕还林工程建设成效. *Hubei Forestry Science and Technology* **43,** 74-77 (2014).

175. Liu, Y., Wang, Y., Wang, H., Wang, Y., Sun, S., Qin, F. & Liu, L. Function and value assessment of soil and water conservation under six patterns of the Grain to Green Program in the Three Gorges Reservoir Area of the Yangtze River. *Science of Soil and Water Conservation* **12,** 50-58 (2014).

176. Wang, H., Xi, Y., Cheng, Q. & Cui, G. 三峡库区退耕还林模式的创建运用与评价. *Forestry Science, Technology and Development 林业科技开发* **21,** 79-82 (2007).

177. Wang, J. & Zhang, T.远安县退耕还林工程发展现状及对策研究. *Journal of Green Science and Technology* **1,** 149-150 (2015).

178. Pan, L., Shi, Y., Xiong, Y., Wang, Z., Xiang, Z. & Ma, D. 秭归县退耕还林水源涵养效益计量 Quantitative evaluation on water conservation benefit of converting land for forest in Zigui County. *Hubei Forestry Science and Technology* **3,** 1-4 (2006).

179. Song, W. & Yin, H. 中坝村退耕还林效益分析. *Hubei Forestry Science and Technology* **148,** 4-6 (2007).

180. Tan, B., Cui, H., Pan, L., Ma, D. & Wang, X. Analysis on carbon fixation and oxygen release benefit of Returning Farmland to Forest in Zigui County. *Hubei Forestry Science and Technology* **43,** 6-8 (2014).

181. Wang, F. Study on ecological benefit assessment of different vegetation restoration models in Lanlingxi small watershed. *Hubei: Huazhong Agricultural University* 1-78 (2008).

182. Wang, Z., Wang, X., Shi, Y., Pan, L., Yu, X. & Tang, G. Effects of soil and water conservation of the conversion of farmland to forest in Zigui County of the Three Gorges Reservoir region. *Science of Soil and Water Conservation* **5,** 68-72 (2007).

183. Wang, X., Wan, F., Cui, H., Pang, H., Pan, L. & Tang, H. Located monitoring of soil and water conservation benefits of different reforestation patterns in Three Gorges Reservoir Region. *Hubei Forestry Science and Technology* **43,** 1-4 (2014).

184. Wu, Q. The benefit evaluation of converting farmland to forest in Zigui. *Beijing: Beijing Forestry University* 1-95 (2011).

185. Wu, D., Huang, Z., Xiao, W. & Zeng, L. Control of soil nutrient loss of typical reforestation patterns along the Three Gorges Reservoir area. *Environmental Science* **36,** 3825-3831 (2015).

186. Zeng, L., Xiao, W., Huang, Z., Lei, J., Wang, P. & Tan, B. Relationship between environmental factors and soil erosion with different patterns of returning farmland to forest in the Three Gorges Reservoir Area. *Resources and Environment in the Yangtze Basin* **23,** 146-152 (2014).

187. Zhou, W. 湖南慈利县退耕还林工程生态效益评价研究. *Beijing Forestry University (Master's Thesis)* 1-74 (2011).

188. Huang, Z. & Guo, X. Discussion on problems and countermeasures of the follow-up project of returning farmland to forest in Xupu County. *Hunan Forest Science and Technology* **40,** 92-94 (2013).

189. Peng, W., Zhang, K. & Yang, Q. Research on soil property dynamics in retired farmland in the Loess sloping land. *Journal of Arid Land Resources and Environment* **20,** 153-158 (2006).

190. Bai, X., Hu, Y., Zeng, D., Jiang, Z. Effects of farm land afforestation on ecosystem carbon stock and its distribution pattern in semi-arid region of Northwest China. *Chinese Journal of Ecology* **27,** 1647-1652 (2008).

191. Bai, X. Effect of afforestation of agricultural land on carbon stocks and allocation pattern in semi-arid sandy region. *Gansu: Gansu Agricultural University* 1-41 (2008).

192. Hu, Y., Zeng, D. & Jiang, T. Effects of afforested poplar plantations on thes tock and distribution of C, N, P at Keerqin Sandy Lands. *Acta Ecologica Sinica* **29,** 4206-4214 (2009).

193. Li, S. SD-based study of the optimized model of conversion of farmland to forests on typical sites. *Journal of Beijing Forestry University* **28,** 22-28 (2006).

194. Guo, R., Lian, H. & Zhao, Y. Effect evaluation of Conversion Farmland to Forest (Grass) Land in Zhuozi County. *Journal of Inner Mongolia Agricultural University* **26,** 12-16 (2005).

195. Cong, W. Effects of afforestation on soil organic carbon content and composition characteristics in Northeastern China. *北京:中国农业大学* 1-82 (2014).

196. Wang, C., Liu, Y., Shao, B. & Zhao, J. Quantifying the soil carbon changes following the afforestation of former arable land. *Journal of Beijing Forestry University* **29,** 112-119 (2007).

197. Wang, C., Shao, B. & Wang, R. Carbon sequestration potential of ecosystem of two main tree species in Northeast China. *Acta Ecologica Sinica* **30,** 1764- 1772 (2010).

198. Chang, Z. 辽宁省巩固退耕还林成果长效机制的探讨. *Journal of Liaoning Forestry Science & Technology* **3,** 41-42 (2009).

199. Yang, H., Zhang, J., Zhang, Y., Gao, W., Wang, W., Tao, Y. & Bai, R. Influences of different Patterns in converting cropland to forestland Project on surface run- off and soil physico- chemical character. *Jilin Forestry Science and Technology* **36,** 29-33 (2007).

200. Cai, L., Xu, Y., Yu, W., Hu, B. & Wu, Y. Effects of continuous planting popular on the soil nutrient contents, microbial biomass, and enzyme activities in lower reaches of Liaohe River Plain, Northeast China. *Chinese Journal of Ecology* **32,** 337-343 (2013).

201. Zhao, J., Zhao, C., Zhang, Q., Wang, Z., Yang, J., Dou, J. & Feng, X. The measures to consolidate the achievements of; and conversion project in Ningxia. *Journal of Agricultural Sciences* **31,** 59-62 (2010).

202. Niu, Z. 西吉县退耕还林工程生态效益探讨. *Modern Agricultural Science and Technology* **20,** 169, 173 (2014).

203. Gao, G., Li, W., Zhou, X., Shi, C. & Li, S. Hydrological effects of different species arrangement models of converted farmland to forest in Beichuanhe Watershed. *Journal of Soil and Water Conservation* **20,** 11-15 (2006).

204. Ma, Z., Zhou, X. & Shi, C. Improvement of soil physical and chemical properties after "Conversion of Farmland to Forest" in alpine areas. *Forest Resources Management* 72-76 (2008).

205. Zhao, C., Wang, S., Sun, G., Yang, Q., Yang, N., Zhang, W. & Dong, X. Study on soil erosiveness in the area of Returning Farmland to Forest in Huangshui River Watershed of Qinghai Province. *Forest Resources Management* **2,** 73-77 (2010).

206. Wang, J. 青海省海东地区退耕还林的调查与思考. *Journal of Anhui Agricultural Sciences* **41,** 7547-7548 (2013).

207. Zhao, C., Dong, X., Xin, W., Wang, S. & Yang, Q. Effect on soil and water conservation from different models of Conversion of Farmland to Forest in Huangshui River at Qinghai. *Journal of Soil and Water Conservation* **23,** 26-29 (2009).

208. Ma, H. Carbon density and its distribution characteristics of the main vegetation ecosystem in the northern of Shaanxi. *Shaanxi: Northwest A and F University* 1-59 (2013).

209. Wang, X. 陕西渭北刺槐林分生长收获模型研究. *Shaanxi: Northwest A and F University* 1-65 (2001).

210. He, J. 汉江流域安康段退耕还林对水质保护作用评估研究. *Shaanxi Journal of Agricultural Sciences* **61,** 54-56 (2015).

211. Cao, S., Chen, L. & Yu, X. Impact of China's Grain for Green Project on the landscape of vulnerable arid and semi-arid agricultural regions: a case study in northern Shaanxi Province. *Journal of Applied Ecology* **46,** 536-543 (2009).

212. Feng, D., Sun, B., Guo, J. & Li, R. Research community succession and α species diversity after Conversion from Cropland into Forest. *Journal of Anhui Agri. Sci.* **37,** 15320-15322 (2009).

213. Feng, D. The research about the ecological benefit value monitoring and evaluation Project to Forest in Ansai District. *Beijing: Beijing Forestry University* 1-90 (2010).

214. Gao, G., Li, D., Jia, J., Hu, W. & Liu, G. Research on soil fertility of different species arrangement models in converted farmland land. *Journal of Arid Land Resources and Environment* **21,** 104-107 (2007).

215. Han, X., Yang, G., Tong, X., Feng, Y., Ren, G., Zhao, F. & Du, Y. Soil carbon and nitrogen sequestration under several different forest lands converted by farmland in Loess hilly area. *Journal of Agro-Environment Science* **31,** 1172-1179 (2012).

216. Li, T., Ren, B., Wang, D. & Liu, G. Spatial variation in the storages and age-related dynamics of forest carbon sequestration in different climate zones – evidence from black locust plantations on the Loess Plateau of China. *PLoS ONE* **10,** e0121862 (2015).

217. Liu, Y., Wang, Q., Yu, G., Zhu, X., Zhan, X., Guo, Q., Yang, H., Li, S. & Hu, Z. Ecosystems carbon storage and carbon sequestration potential of two main tree species for the Grain for Green Project on China's hilly Loess Plateau. *Acta Ecologica Sinica* **31,** 4277-4286 (2011).

218. Ma, X., Jiao, J., Bai, W., Jiao, F. & Wen, Z. Contribution of soil nutrient in abandoned lands to vegetation restoration in hilly and gully regions on the Loess Plateau. *Acta Agriculturae Boreali-occidentalis Sinica* **25,** 328-335 (2005).

219. Sun, C. Effect of vegetation restoration on soil carbon sequestration and carbon fractions in northern Shaanxi. *Shaanxi: Northwest A and F University* 1-96 (2014).

220. Tong, X., Han, X., Yang, G., Feng, Y., Ren, G., Liang, A. & Kang, D. Carbon management index as an indicator for changes in soil organic carbon pool under conversion from cropland to forestland. *China Environmental Science* **33,** 466-473 (2013).

221. Xu, M., Wang, Z., Zhang, J. & Liu, G. Response of soil organic carbon sequestration to the “Grain for Green Project” in the hilly Loess Plateau region. *Acta Ecologica Sinica* **32,** 5405-5415 (2012).

222. Zhang, Y., Liang, A., Wang, P., Yang, G. & Zhang, X. Effect of different vegetation restoration models on soil fertility in Zhifanggou Watershed in gully region in Loess Region. *Acta Agriculturae Boreali-occidentalis Sinica* **9,** 114-118 (2010).

223. Zhang, X., Chen, J. & Zhao, Y. Impact of China's Grain for Green Project on the ecological environment in northern Shaanxi Province. *Beijing Agriculture* **9,** 48-51 (2011).

224. Zhao, F., Han, X., Yang, G., Tong, X., Kang, L. & Du, Y. Changde characteristics of density of soil organic carbon and nitrogen under land shifted into forestland in hilly Loess region. *Research of Soil and Water Conservation* **19,** 43-47 (2012).

225. Bai, H., Lan, T. & Wang, D. Grain for Green Program in Baota District: status and future trend. *Shaanxi Forest Science and Technology* **107,** 100-103 (2009).

226. Li, Z., Guo, S., Zhang, F. & Zou, J. Effects of apple orchard converted from cropland on C and N storages in terrestrial system of slopping cultivated land in the Loess gully regions. *Plant Nutrition and Fertilizer Science* **17,** 919-924 (2011).

227. Guo, M. A Study on response of the soil micromorphology characteristics to Returning Farmland to Forest in Heyang Area. *Shaanxi: Shaanxi Normal University* 1-71 (2008).

228. Pang, J., Guo, M., Qiu, H. & Guo, G. Effect of "Grain for Green" on micromorphological features of the soil in east Guanzhong Region. *Acta Pedologica Sinica* **46,** 210-217 (2009).

229. Dong, L. & Zheng, F. Effects land-use types on soil microbial characteristics and carbon density in the loessial hilly-gully region. *Journal of Shaanxi Normal University (Natural Science Edition)* **37,** 88-94 (2009).

230. Ren, J. Study on carbon sink compensation of returning farmland to forest change in Mizhi county of Shaanxi Province. *Shaanxi: Northwest A and F University* 1-47 (2013).

231. Li, X., Li, Y., Yu, H., Zhang, Y. & Guo, Z. Spatial changes in soil CO_2 emission from re-forested hillslopes on the Loess Plateau: a geomorphic control. *Bulletin of Plant Nutrition and Fertilizer* **21,** 1217-1224 (2015).

232. Hu, J. The effect of soil and water conversion and ecological effect of water and fertilizer on soil under different vegetation restoration. *Shaanxi: Northwest A and F University* 1-59 (2007).

233. Zhang, Y., Gou, S., Han, E. & Han, G. Effects of different models used in land conversion on tree growth and soil nutrients. *Shaanxi Forest Science and Technology* **4,** 15-17 (2007).

234. Zhang, X., Yang, G., Wang, D., Feng, Y. & Ren, G. Effect of different vegetation restoration models on soil microbial characters in the gully region of Loess Plateau. *Journal of Northwest A & F University (Natural Science Edition)* **36,** 149-159 (2008).

235. Hu, X. Major measures applied in the Grain for Green Project in Shanyang. *Shaanxi Forest Science and Technology* **2,** 47-48 (2013).

236. Guo, J., Li, J., Liu, T., Sun, B., Liang, Z. & Zhao, Y. Effect of Conversion to Forestland on soil erosion and land use/ cover in Wuqi County. *Research of Soil and Water Conversion* **20,** 1-6 (2013).

237. Moberg, J. & Persson, M. The Chinese Grain for Green Program – assessment of the land reform's carbon mitigation potential. Master of Science thesis in the Master

Degree Program, Sustainable Energy Systems, Chalmers University of Technology, Göteborg, Sweden (2011).

238. Chen, X., Xing, E., Dong, Z., Guo, J. & Li, J. Effects on carbon storage by Conversion of Cropland to Forest in Wuqi County. *国际沙棘研究与开发* **4,** 32-37 (2014).

239. Cheng, J., Bai, C., Liang, W. & Sun, B. Study on soil water availability and water storage capacity in the area of Upland Conversion Program - case study in Chaigou Watershed of Wuqi County, ShaanXi Province. *Shaanxi Forest Science and Technology* **4,** 1-3 (2005).

240. Dong, L., Chen, Y. & Li, X. Effects of the Returning Farmland to Forests on content of water stable soil aggregates and the nutrients in Wuqi County. *Scientia Silvae Sinicae* **50,** 140-146 (2014).

241. Dong, L., Ma, X., Hu, D. & Qian, Y. Monofractal and multifractal characteristics of soil particle size distribution in sample sites of Grain for Green in Wuqi County. J*ournal of Arid Land Resources and Environment* **29,** 111-115 (2015).

242. Guo, J. Research on monitoring and benefit evaluation of Project for Conversion of Cropland to Forestry in Wuqi County. *Beijing: Beijing Forestry University* 1-186 (2009).

243. Xing, J., Guo, J., Zhao, X. & Lan, D. Research on soil physical properties after conversion of cropland to forest in Loess hilly region - taking examples of Wuqi County. *Journal of Inner Mongolia Agricultural University* **31,** 41-46 (2010).

244. Yu, M., Sun, B., Hu, S., Wang, H. & Zhao, Y. Modeling the degree of coupling and interaction between forest structure and ecological function in a grain for green project, Shanxi, China. *Acta Ecologica Sinica* **34,** 4991-4998 (2014).

245. Chao, S. Evaluation on the ecological and economic benefits of the conversion of cropland to forest (grassland) in Weibei Rainfed highland - A case study in Xiaoqiu town of Hanjiayuan plateau of Yaozhou district. *Shaanxi: Shaaxi Normal University* 1-68 (2008).

246. Hong, Y. & Li, X. Cultivated land and food supply in China. *Land Use Policy* **17,** 73-88 (2000).

247. Li, H., Fan, S., Zhang, G., Zhang, S. & Zhou, Z. Characteristics of soil water-holding and soil porosity under different tree species after Conversion of Cropland to Forest in the Loss Hilly Region. *Bulletin of Soil and Water Conservation* **30,** 27-30 (2010).

248. He, Y., Fei, S., Jiang, J., Chen, X., Yu, Y., Zhu, W. & Wang, S. Researches on the vegetation recovery of some bamboo species for Conversion of Land to Forest in southern Sichuan. *Journal of Sichuan Forestry Science and Technology* **27,** 7-13 (2006).

249. Zhang, D. Assessment on soil and water conversion function of de- farmed bamboo plantation in south Sichuan Province. *Beijing: Chinese Academy of Forestry* 1-161 (2012).

250. Zhang, D., Fan, S., Cai, C., Liu, G. & Liu, Y. Soil aggregates of returning farmland to different bamboo forests in southern Sichuan Province. *Scientia Silvae Sinicae* **49,** 27-32 (2013).

251. Huang, C., Zhang, J., Deng, Y. & Yang, W. Carbon storage and allocation patterns of young forests converted by farmland. *Journal of Soil and Water Conservation* **21,** 130-133 (2007).

252. Li, J., Huang, C. & Zhang, G. Density, storage and spatial distribution of carbon in *Pleioblastus amarus* forest returned from farmland. *Journal of Zhejiang Forestry Science and Technology* **26,** 1-5 (2006).

253. Liu, Z., Zhu, T. & Zhang, J. Analysis on communities of soil microbes under different models of forest rehabilitation. *Journal of Nanjing Forestry University (Natural Sciences Edition)* **29,** 45-48 (2005).

254. Tang, J., Li, X., Lai, Y., Feng, S., Wang, P. & Huang, C. Spatial and temporal patterns of bamboo carbon storage in forest land converted from farmland in Hongya， Sichuan. *Scientia Silvae Sinicae* **47,** 1-7 (2011).

255. Luo, L., Hu, T. & Wan, X. Dynamic variation of physical and chemical properties of soil in different patterns of returning to forest. *Journal of Zhejiang Forestry Science and Technology* **26,** 18-22 (2006).

256. Song, X. The analysis of the sustainability of Grain for Green Project-a case study of Sichuan Tian Quan county. *Jiangsu: Nanjing Forestry University* 1-63 (2010).

257. Wan, X., Hu, T., Zhang, J., Li, X. & Gong, Y. Vegetation restoration of hillside fields after Conversion of Farmland to Forests. *Scientia Silvae Sinicae* **41,** 191-194 (2005).

258. Yang, D. Studies on dynamics of soil nutrition in two reforested patterns from farmland in Sichuan Tianquan. *Sichuan: Sichuan Agricultural University* 1-47 (2005).

259. Ke, Y., Yang, H., Wang, X., Hu, T., Liu, Y. & Zhu, B. Effect of Returning Cropland to Forestland on N_2O emissions from sloping purple soil. *Journal of Agro-environment Science* **34,** 1398-1406 (2015).

260. Chen, X., Zhang, X., Zhang, Y. & Wan, C. Carbon sequestration potential of the stands under the Grain for Green Program in Yunnan Province, China. *Forest Ecology and Management* **258,** 199-206 (2009).

261. Weyerhaeuser, H., Wilkes, A. & Kahrl, F. Local impacts and responses to regional forest conservation and rehabilitation programs in China's northwest Yunnan province. *Agricultural Systems* **85,** 234-253 (2005).

262. Shen, S., Wilkes, A., Qian, J., Yin, L., Ren, J. & Zhang, F. Agrobiodiversity and biocultural heritage in the Dulong Valley, China. *Mountain Research and Development* **30,** 205-211 (2010).

263. Wang, Z. 普洱市实施退耕还林工程效益分析. *Inner Mongolia Forestry Investigation and Design* **37,** 134-136 (2014).

264. Chen, Q., Chang, E., Bi, B., Li, P., Yin, A., Liu, Y. & Nong, S. Study on conservation of soil and water in three different forestation modes in karst area of southeastern of Yunnan. *Journal of Soil and Water Conservation* **20,** 1-4 (2006).

265. Yuan, B. Research in vegetation restoration and soil remediation of Grain for Green - Taking Qingyang of Gansu Province as an example. *Hubei: Huazhong Agricultural University* 1-53 (2006).

266. Yuan, B., Qin, T., Fan, Z., Wang, W. & Huang, H. Research on effect of different "Conversion of Farmland to Forest" practices on soil remediation - take Qingyang City of Gansu Province as an example. *Forest Resources Management* **6,** 51-54 (2005).

267. Wu, C., Wei, X., Shi, H., Zhuang, J. & Lai, J. 退耕还林尾叶桉模式和任豆模式的制备调查. 安徽农业科学 **36,** 2765-2767 (2008).

268. Long, J., Deng, Q., Jiang, X., Li, Y. & Yao, B. Effects of landuse types on restoration of soil quality on karst rocky desertification region in Guizhou Province. *Acta Ecologica Sinica* **25,** 3188-3195 (2005).

269. Pan, L., Tang, W., Xiao, W., Shi, Y., Zeng, L. & Huang, Z. Woodland hydrological effects of converting cropland to forest in the three gorges reservoir region. *Bulletin of Soil and Water Conservation* **32,** 103-106 (2012).

270. Liu, J., Qin, F., Yue, Y., Wang, C. & Sheng, Y. Evaluation of ecological benefits on restoration in Yijinhuoluo sandstorm area of Inner Mongolia. *Research of soil and water conversion* **20,** 104-107 (2013).

271. Cao, S., Chen, J., Chen, L. & Gao, W. Impact of Grain for Green Project to nature and society in north Shaanxi of China. *Scientia Agricultura Sinica* **40,** 972-979 (2007).

272. Liu, Z. Coupling relationship between ecology effect and economy effect of returning farmland into forest or grasses. *Shaanxi: Northwest A and F University* 1-57 (2007).

273. Liang, A., Han, X., Zhao, F., Ren, G. & Yang, G. Dynamics of soil carbon and nitrogen stocks following afforestation in gully regions of Loess Plateau, China. *Transactions of the Chinese Society of Agricultural Engineering* **30,** 148-157 (2014).

274. Yu, Z. Study on benefit of plantation - medicine intercropping models in land conversion project of Shanyang County. *Shaanxi: Northwest A and F University* 1-36 (2007).

275. Cai, B. 四川朝天: 核桃产业成为退耕还林的动力源. *中国经贸导刊* **9,** 52 (2015).

276. Li, S., Zhu, T. & Liu, Z. Evaluation on effect of Grain-for-Green models on soil biological properties. *Bulletin of Soil and Water Conservation* **33,** 129-135 (2013).

277. Liang, J., Zhang, J. & Yang, D. The dynamic of soil available nitrogen under several patterns of the Converting Lands into Forests in Hongya, Sichuan. *Journal of Sichuan Agricultural University* **23,** 48-51 (2005).

278. Liu, Z. Study on effect of different models of conversion of cropland to forest on soil microbes and enzyme activities. *Sichuan: Sichuan Agricultural University* 1-70 (2005).

279. Liu, Z., Zhu, T. & Zhang, J. Characteristics of soil microbes for two models of forest rehabilitation. *Journal of Soil and Water Conservation* **20,** 132-135 (2006).

280. Xu, Y., Zhang, J. & Liang, J. Development research on soil physical and chemical properties in four de-farming and reafforestation patterns. *Journal of Sichuan Agricultural University* **25,** 294-299 (2007).

281. Wang, H., Chen, Z., Li, X. & Wang, H. Study on the soil nutrient and tree and grass nutrient content cycle of different forest and grass system in Converting Agricultural Lands to Trees. *Research of Soil and Water Conservation* **13,** 124-127 (2006).

282. Yang, D., Luo, C., Guan, Y. & Liang, J. The dynamic of soil nutrient under forest and grass composite pattern in area of conversion of farmland to forests. *Scientia Silvae Sinicae* **43,** 101-105 (2007).

283. Su, C. & Shang, Y. Study on soil anti-scourability under different converting cropland into forest patterns. *Journal of Henan Agricultural Science* **43,** 57-63 (2014).

284. Gao, G., Zhou, X., Shi, C., Li, W. & Li, S. Soil effects of converted farmland in remote hilly areas in Beichuanhe Watershed. *Agricultural Research in the Arid Areas* **26,** 206-211 (2008).

285. Wang T, Guo J, Sun B, Li J, Wang Q & Ren, Z. The Research on Soil Physical Properties of Farming-Pastoral Zone after Cropland Conversion to Forest - Taking Zhuozi County, Wulanchabu City, Neimengu as the Example. *Jour of Fujian Forestry Sci and Tech* **37,** 17-21 (2010).

286. China Bird Report Ed. The CBR checklist of birds of China, v3.0. Available on 31 December, 2013 (2013).
